# A comprehensive view of the web-resources related to sericulture

**DOI:** 10.1093/database/baw086

**Published:** 2016-06-15

**Authors:** Deepika Singh, Hasnahana Chetia, Debajyoti Kabiraj, Swagata Sharma, Anil Kumar, Pragya Sharma, Manab Deka, Utpal Bora

**Affiliations:** ^1^Bioengineering Research Laboratory, Department of Biosciences and Bioengineering, Indian Institute of Technology Guwahati, Guwahati, Assam 781039, India; ^2^Centre for Biological Sciences (Bioinformatics), Central University of South Bihar (CUSB), Patna 800014, India; ^3^Department of Bioengineering & Technology, Gauhati University Institute of Science & Technology, Gauhati University, Guwahati, Assam 781014, India; ^4^Centre for the Environment, Indian Institute of Technology Guwahati, Guwahati, Assam 781039, India; ^5^Mugagen Laboratories Pvt. Ltd, Technology Incubation Centre, Indian Institute of Technology Guwahati, Guwahati, Assam 781039, India

## Abstract

Recent progress in the field of sequencing and analysis has led to a tremendous spike in data and the development of data science tools. One of the outcomes of this scientific progress is development of numerous databases which are gaining popularity in all disciplines of biology including sericulture. As economically important organism, silkworms are studied extensively for their numerous applications in the field of textiles, biomaterials, biomimetics, etc. Similarly, host plants, pests, pathogens, etc. are also being probed to understand the seri-resources more efficiently. These studies have led to the generation of numerous seri-related databases which are extremely helpful for the scientific community. In this article, we have reviewed all the available online resources on silkworm and its related organisms, including databases as well as informative websites. We have studied their basic features and impact on research through citation count analysis, finally discussing the role of emerging sequencing and analysis technologies in the field of seri-data science. As an outcome of this review, a web portal named SeriPort, has been created which will act as an index for the various sericulture-related databases and web resources available in cyberspace.

**Database URL:** http://www.seriport.in/

## Introduction

More than 50 years have passed since the time when the term ‘database’ was coined. However, it was only during the massive digitalization of many resources like archives of music, books, etc. in the 1990s that the same term started reflecting its primary usage in today’s world as a data organizational model (1). During these years, the databases have been empowered to retrieve and filter data in various ways. Integration of these databases with biology has brought digital revolution to life science. The amalgamation of biology with information technology for data dissemination and statistics for data analytics has led to the development of some highly successful databases like GenBank, RCSB Protein Data Bank (PDB), etc. (2). Now, there are databases in almost every field of biology ranging from diseases, whole organisms, taxonomy, bioactive products, etc. making them indispensable for the researchers (3–6). One of the research fields in which databases are being constructed actively is ‘Sericulture’.

Silkworms and their respective host plants are the key players of sericulture and silk is its prime yield. Sericulture has been in practice much prior to the Silk Road era in ancient Indian and Chinese civilizations and helped in the enrichment of human endeavors in art and culture (7). *Bombyx mori*, *Antheraea assamensis*, *A. mylitta* and many other silkworms are responsible for the production of silk varieties like mulberry silk, muga silk, tasar silk, etc. for traditional and commercial usage. Researchers also developed mutants of these organisms for improving silk quality and quantity, understanding their physiology and exploiting them as bioreactors for recombinant proteins (8–10). Similarly, the host plants of silkworms are studied not only due to their importance as a survival requisite for silkworms but also for several unconventional benefits like production of biodiesel, medicinal applications, etc. (11, 12). Apart from these, other members of a silkworm’s ecosystem like pests and pathogens which threaten the existence of the silkworms are also researched for the development of treatment or pest-control strategies, host–pathogen interaction studies, etc. (13, 14).

**Figure 1.**
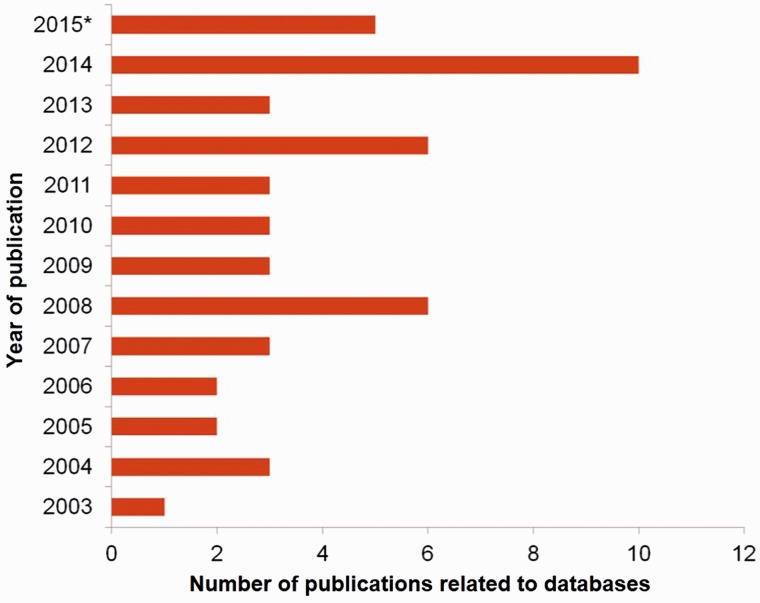
Number of publications on seri-related databases from the year 2003 to 2015* where (*) represents 2015-continued year.

The need of databases in sericulture field cannot be emphasized more. First, numerous organisms are involved in this field and scientists have uncovered minuscule information about most of them while some are yet to be identified. Second, the data that are generated in this field are of dissimilar type. Each data ranging from nucleotide and protein sequence to gene maps, expression profiles and biomaterials, is unique and vital. Third, the amount of data generated is huge due to the fast-evolving techniques of sequencing, analysis, imaging, etc. Especially, the sequencing techniques have progressed beyond shotgun-sequencing to more quick and efficient next generation sequencing (NGS), chromatin immuno-precipitation sequencing or ChIP-Seq, etc. which produce millions of sequence data at a go (15, 16). Till now (2003–present year), the total number of published databases in this seri-bioresource field is 50 out of which 27 were created in the last five years (Figure 1).

Therefore, in order to boost the research in this field, each type of data generated by scientists must be cross-checked for reliability and then archived in separate digital databases to create a helpful online space for users. These databases must be made openly accessible to others and equipped with analytical tools. This will promote better research and facilitate development of improved scientific strategies in this field.

**Figure 2.**
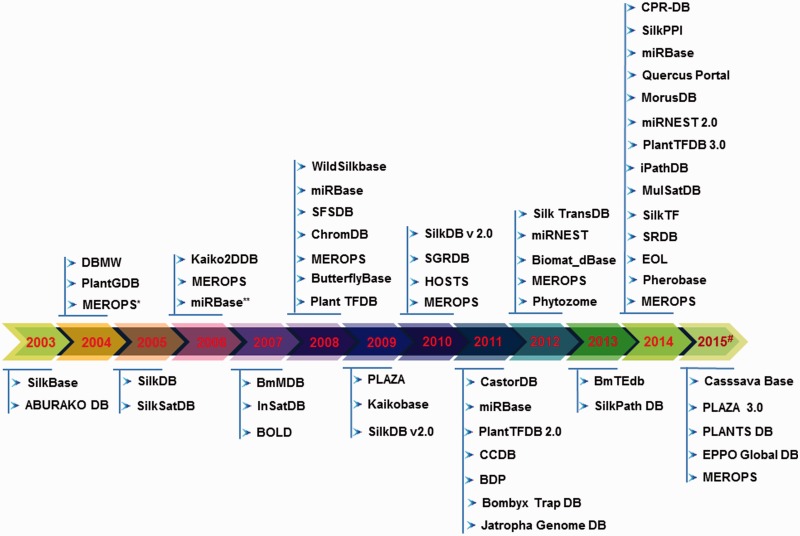
Timeline of the existing seri-databases from the year 2003 to 2015# generated using respective publication in the literature and database creation year from websites, where (#) represents 2015-continued year; (*) indicates database first published in 1999 and its updated versions considered from period 2003–2015; (**) indicates the same database with updated information.

**Figure 3.**
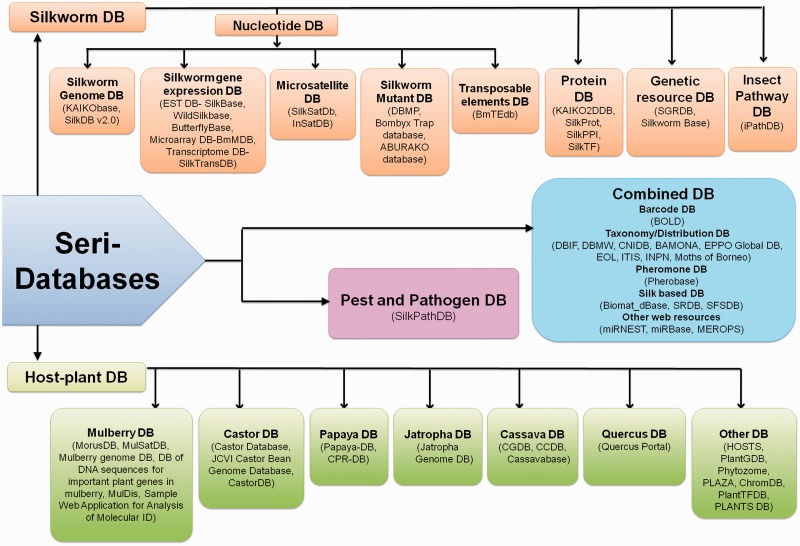
Schematic representation of Seri-databases classified into four categories- Silkworm Databases-20 No.s, Host Plant Databases-23 No.s, Pest and Pathogen Databases- 01 No., Combined Databases-17 No.s.(Abbreviations- SilkDB: Silkworm Knowledgebase, EST DB: Expressed Sequence Tag Database, BmMDB: Bombyx mori Microarray Database, SilkTransDB: Silkworm Transcriptome Database, SilkSatDb: Silkworm Microsatellite Database, DBMP: Database of Bombyx mutant photographs, BmTEdb: Transposable elements database for *B. mori*, SilkProt: Annotated protein database of silkworm, SilkPPI: Silkworm Protein–Protein Interaction database, SilkTF: Silkworm Transcription Factor Database, SGRDB: Silkworm Gene Resources database, iPathDB: Insect Pathway Database, MorusDB: Morus Genome Database, MulSatDB: Mulberry Microsatellite Database, CastorDB: A comprehensive knowledgebase database for *R. communis*, Papaya-DB: Papaya Genomic Resources Online, CPR-DB: Papaya Repeat Database, CGDB: Cassava Genome Database, CCDB: Chinese Cassava Genome Database, HOSTS: a Database of the World's Lepidopteran Host plants, PlantGDB: Resources for Comparative Plant Genomics, ChromDB: The Chromatin Database, PlantTFDB: Plant Transcription Factor Database, SilkPathDB: Silkworm Pathogen Database, BOLD: Barcode of Life Data System, DBIF: Database of Insects and their Food Plants, DBMW: Database of Butterflies and Moth of the World, CNIDB: Common Names of Insects Databases, BAMONA: Butterflies and moths of North America, EOL: Encyclopedia of Life, ITIS: Integrated taxonomic information system, SRDB: Spatio-temporal database of Silk Road, SFSDB: Silk Fabric Specification Database, miRNEST: An integrative microRNA resource, miRBase: The microRNA database, MEROPS: the peptidase database).

**Figure 4.**
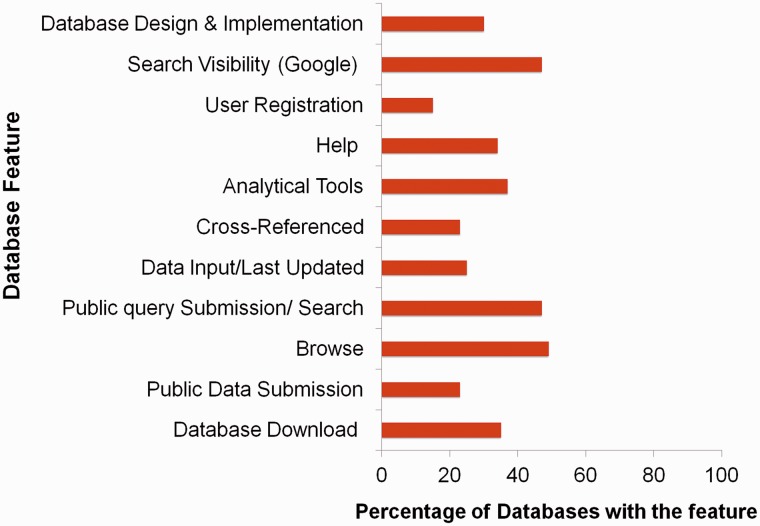
Percentage distribution of various features across seri-databases.

In this review, we have collated the available databases on sericulture from 2003 till now (Figure 2) and categorized them based on the type of datasets (Supplementary Table S1). Our search attempt has led to identification of 61 databases which comprise of genome, proteome, transcriptome and other data of silkworms, host plants, pest and pathogens, etc. The databases have been briefly discussed here and schematically depicted in Figure 3. While the prime requisite of a database is to provide good quality data, it must also have an optimal web interface with integral features like search, browse, data download, etc. Quality of data can be maintained by proper data deduction methods. For example, the reliability of a transcriptome dataset can usually be depicted in the depth of sequencing. The quality can also be enhanced by regular data update and cross-referencing, simultaneously removing redundancy in the datasets. Also for a web interface, its navigation features like browser allows thorough scanning of complete datasets and search engine helps a user to find the data of interest without the hassles of browsing the whole dataset. Another integral part of a database is the data download/upload option. Sometimes, huge datasets like genome or transcriptome require analyses that are not possible over the internet. In such instances, data download feature becomes really helpful. Similarly, data upload feature enables a researcher to upload their findings into a database (submitted data should always be subjected to curation by the database administrator), concurrently increasing the quantity of data. Again, user registration is not a necessary feature, but can be a useful addition to any database. Depending on the design of the registered user’s interface, this feature can help a user to keep track of his or her submitted data or data of interest. Taking these features into account (Figure 4), a comparative table has been created, depicting their presence or absence (Table 1). Towards the end of the review, we have discussed potential scope and impact of these databases as well as contribution of technology to the field of sericulture and related areas. Furthermore, we have designed a user-friendly and dynamic web portal named ‘SeriPort’ to accommodate all the available databases as well as web-resources related to sericulture field. The portal can be accessed at http://www.seriport.in/. This review will be helpful for the researchers and other enthusiasts in the field of sericulture as well as broader area of entomology.

**Table 1.** Comparative features of Seri-databases (Data Search on 11 March 2016)

Name	Website design & implementation	Database download enabled	Public data submission	Browse	Public query submission/search	Data input	Cross-referenced	Associated with online analytical tools	Help	User registration	Search visibility (Google)	References	Comments
						Last Updated
Silkworm databases													
Silkworm genome databases													

SilkDB (v2.0)	-MySQL-Navigated by GBrowse	✓ (via ftp)	✗	✓ (GBrowse, SCB tool)	✓ (Quick Search, etc.)	✗	✓	✓ (SilkMap, BLAST, Wego, Clustal W, Cap3, BL2SEQ, EMBOSS, WISE2)	✓	✗	Fair	(24)	http://www.silkdb.org
						(2009)							

Kaikobase	-Written in Javascript-**Search:** PostgreSQL version 8.2.1-**Gene Viewer:** HMMER version 2.1.1, ProfileScan version 2.2, PSORT version 6.4, SOSUI version 1.0, MOTIF, and InterProScan version 4.3.1 (data version 14.0)- GBrowse	✓ (Through **GBrowse** and in different formats- PNG, GFF, FASTA, zip, text)	✓ (can upload and share custom tracks in GBrowse)	✓ (GBrowse, UTGB and GeneViewer)	✓ (Keyword and Position Search, Scaffold Sequence Search)	✗	✗	✓ (KAIKOBLAST, KAIKOGAAS, GBrowse)	✓	✗	Fair	(23)	http://sgp.dna.affrc.go.jp/KAIKObase/ (version 3.2.2: 13/05/2013)
						07/06/2013							

Silkworm gene expression databases													

SilkBase	NA	✓ (cDNA and EST for *Bombyx mori* and *Samia cynthia* ricini in FASTA)	✗	✓ (Libraries tab)	✓ (Different Search Bar- Keyword, gene model, genome position, ESTs, etc.)	✓	✓	✓ (All variations of BLAST)	✗	✗	Fair	(29)	http://silkbase.ab.a.u-tokyo.ac.jp/cgi-bin/index.cgi
						16/03/2015							

WildSilkbase	MySQL and PHP, Apache web server, Fedora Linux system	✓ (EST Sequence data, EST annotation data)	✗	✓	✓ (3 searches-Keyword Search, Homolog Finder and SSR Finder)	✗	✗	✓ (BLAST, Homolog Finder, SSR Finder)	✓	✗	Fair	(30)	http://www.cdfd.org.in/wildsilkbase/

Butterfly Base	**PostgreSQL** with a customized version of the **PartiGene** schema	✓ (FASTA)	✓	✓ (Genome Browse Under Development)	✓ (Text search, BLAST search, etc.)	✓	✗	✓ (NCBI-BLASTALL, PSI-BLAST and WU-BLAST-driven MS-BLAST, prot4EST)	✓	✗	Poor	(31)	http://www.butterflybase.org/ Currently link is not working (features are based on publication)

BmMDB	MySQL, **Php scripts:** database query, generate HTML or heat map picture to display the query results.	✗	✗	✓ (Browse Raw data, Browse Tissue specific genes)	✓ (Search by Probe ID, Search by BLAST)	✗	✓	✓(BLAST)	✗	✗	Fair	(33)	http://www.silkdb.org/microarray/
SilkTransDB	Gbrowse	✓ (PNG, SVG, FASTA, GFF)	✓ (can upload file in Gbrowse option)	✓ Gbrowse	✓ (BLAST Search)	✗	✓	✓ (BLAST-blastn, tblastn, tblastx)	✓	✗	Fair	(34)	http://124.17.27.136/gbrowse2/

Microsatellite database													
SilkSatDB	MySQL, PHP, Apache web server	✗	✗	✓	✓	✗	✗	✓ (SSRF, AutoPrimer)	✗	✗	Fair	(38)	http://www.cdfd.org.in/silksatdb
						13/10/2004							

InSatDB	MySQL, PHP, Apache web server	✓ (.csv files)	✗	✓	✓ (multi-option query sheet)	✗	✗	✓ (Primer3)	✓(Tutorial)	✗	Fair	(40)	www.cdfd.org.in/insatdb

Silkworm mutant databases													
DBMP	NA	✗ (Photographs of mutants can be accessed)	✗	✓	✗	✗	✗	✗(BLAST associated with full length cDNA DB but currently not functional)	✗	✗	Fair	No publication	http://papilio.ab.a.u-tokyo.ac.jp/genome/ (Connected with Silkbase and Full length cDNA database)

ABURA-KO	NA	✗ (Photographs of mutants can be accessed)	✗	✓	✗	✗	✗	✗	✗	✗	Fair	No Publication	http://cse.nias.affrc.go.jp/natuo/en/aburako_top_en.htm
						07/03/2012							

Bombyx Trap Database	Integrated with Kaikobase	✗ (Photographs of strains can be accessed)	✗	✓	✓ (Word search and Pictorial search options)	✗	✓	✗	✓	✗	Fair	No Publication for database but there is a reference publication (42)	http://sgp.dna.affrc.go.jp/ETDB/ DB is integrated with Kaikobase (23)
						28/02/2011							

Transposable elements databases													
BmTEdb	NA	✓ (FASTA)	✗	✓ (Browse BmTEdb)	✓ (Keyword search)	✗	✗	✓ (BLAST, HMMER, GetORF)	✓	✗	Fair	(43)	http://gene.cqu.edu.cn/BmTEdb/
						07/27/2013							

Silkworm protein databases													
Kaiko2DDB	-**Make-2DDB II** software, HTML-Navigated by **Graphical viewer**	✗ (2D-PAGE Images can be retrieved)	✗	✓	✓ (simple search queries, combined fileds)	✗	✗	✗	✗	✗	Fair	(50)	http://kaiko2ddb.dna.affrc.go.jp/ Integrated with Kaikobase, (23)

SilkProt	NA	✗	✗	✗	✓ (Submit Query)	✗	✗	✗	✗	✗	Poor	No Publication	http://www.btismysore.in/silkprot/ (Lacks proper home page)
SilkPPI	NA	✗	✗	✓	✓	✗	✓	✗	✓	✗	Poor	No publication for DB But the data available is referred in Ref (51)	http://210.212.197.30/SilkPPI/ (Currently link is not functional)

SilkTF	NA	✗	✗	✓ (Browse by id)	✓ (Search by Sequence ID, Domain)	✗	✓	✓(BLAST)	✓	✗	Fair	No Publication	http://www.btismysore.in/SilkTF/

Silkworm genetic resource database													

SGRDB	MYSQL, JAVA, Oracle relational database management system (RDBMS)	–	–	–	–	–	–	–	–	–	Poor	(53)	http://www.naas.go.kr/silkworm/english (Link is not functional)

Silkworm Base	NA	✗	✓ (Publication)	✓	✓ (3 Search options-strain, gene & references)	✓	✓	✗	✗	✓	Fair	No publication for DB	http://www.shigen.nig.ac.jp/silkwormbase/about_kaiko.jsp
						27/04/2015							

Insect pathway databases													
iPathDB	HTML, PHP, CSS and JavaScript, Apache HTTP server, Linux operating system (Redhat 5.6, Raleigh, NC, USA)	✓ (via FTP, Data Sorted by species, Phylogenetic tree, Software: iPathCons, raw data)	✓	✓	✓ (Search by species name, pathway ID or name, Pathway Search)	✓	✓	✓ (iPathCons)	✓	✗	Fair	(54)	http://ento.njau.edu.cn/ipath/

Silkworm host plant databases													
Mulberry databases													
MorusDB	**Server:** Linux Ubuntu Sever 12.04, Apache 2, MySQL Server 5.5, PHP 5.3. **Common gateway interface:** Perl, PHP, C, JavaScriptContent **management system:** Drupal CMS	✓ (via FTP-FTP Downloads, File Browser Downloads)	✗	✓ GBrowse	✓ (Search, Fetch data)	✓	✓	✓ (BLAST, WEGO, Browse GO, Search GO, Genome browser)	✓	✗	Fair	(59, 60)	http://morus.swu.edu.cn/morusdb
						MorusDB v1.0 released (29/08/2014)							
MulSatDB	MySQL relational database, Apache 2.2 web server, Linux system, PHP, HTML and JavaScript	✗	✓	✓	✓ (Search markers-whole genome, EST)	✓	✓	✓ (CMap, Primer3Plus, BLAST)	✗	✗	Fair	(61)	http://btismysore.in/mulsatdb/index.html
						05/02/2014							

Castor databases													

Castor Database	PHP	✗	✗	✓	✓	✗	✗	✗	✗	✗	Poor	No publication	http://www.tnaugenomics.com/castor/index.php (Not accessible on 11-03-2016)
						04/2014							

Other web resources on Castor													

JCVI Castor Bean Genome DB	NA	✓ (via FTP)	✗	✗Gbrowse (Error)	✓ (sequence BLAST search)	✗	✗	✓ (BLAST-blastn, blastp, blastx, tblastn, tblastx)	✗	✗	Fair	No Publication	http://castorbean.jcvi.org/index.shtml (Datasets are out of date)

CastorDB	MySQL, Perl API, Perl, Java, CGI, HTML, Javascript	✓ (text, jnlp file)	✗	✓	✓ (simple, advanced and similarity search)	✗	✗	✓ (BLAST)	✓	✗	Poor	(64)	http://CastorDB.msubiotech.ac.in Currently link is not functional (11/03/2016)

Papaya databases													

Papaya-DB	NA	✗	✗	✓ (Gbrow-se is not function-al)	✓ (Search not-functional)	✗	✓	✗	✗	✗	Fair	Draft genome published in 2008 (120)No Publication for DB	http://www.plantgenome.uga.edu/papaya/ (Not well developed. Most of the links are not working)

CPR-DB	Hosted via ftp	✓ (via FTP-TE, TR, protein sequences can be downloaded)	✗	✗	✗	✗	✗	✗	✗	✗	Fair	(66)	ftp://ftp.cbcb.umd.edu/pub/data/CPR-DB (DB is not accessible.Only download sequence link is available)

Jatropha databases													

Jatropha genome DB	NA	✓ (via FTP)	✗	✓	✓ (Keyword Search, Similarity search)	✓	✗	✓ (BLASTN, BLASTP, TBLASTN)	✗	✗	Fair	(67)	http://www.kazusa.or.jp/jatropha/ (Available in Versions 3.0 and 4.5)
Cassava databases													

CGDB	NA	✓ (BAC End Sequences, Cassava ESTs, Assembled Cassava ESTs, SNPs from physical map and genes)- Right click on dataset name, & select “*Save Link As”*	✗	✓ (Gbrow se is not-function-al)	✗	✗	✗	✓ (BLAST-blast, blastn, blastp, tblastn, tblastx)	✗	✗	Fair	No Publication	http://cassava.igs.umaryland.edu/cgi-bin/index.cgi

CCDB	NA	✓ (FASTA, GFF)	✗	✓ (Genome Browser)	✗	✗	✗	✓ (BLAST)	✓ (Under construction)	✓ (Not functional)	Fair	No Publication	http://www.cassava-genome.cn/
						01/08/2011							

Cassavabase	**solGS:** a web-based tool for genomic selection **Database design:** NA	✓ (via FTP)	✓	✓ GenomeBrowser (Jbrowse)	✓ (Multi- optional search)	✓	✓	✓ (**Sequence Analysis:** BLAST, VIGS Tool, Alignment Analyzer, Tree Browser **Mapping:** Comparative Map Viewer, CAPS Designer, solQTL: QTL Mapping **Molecular Biology:** In Silico PCR **Systems Biology:** SolCyc Biochemical Pathways, Coffee Interactomic Data, SGN Ontology Browser **Breeder Tools:** Breeder Home)	✓	✓	Fair	(121)	http://www.cassavabase.org/ (Manual is provided for users to access the database)
						21/02/2016							

Quercus (Oak) databases													

Quercus portal	NA	✓	✓	✓	✓ (Integrated search-Global search)	✓	✓	✓ (BLAST)	✓ (Overall navigation guide need for easy access)	✓	Fair	–	https://w3.pierroton.inra.fr/QuercusPortal/index.php?p=fmap (Integrated database-Features based on its all databases)
						19/05/2015							
Other generalized plant databases (Not specific for only silkworm host plants but contains some of their information)													

HOSTS	NA	✗	✗	✓	✓(Search & Drill down search-Lepidoptera, Hostplant)	✗	✗	✗	✓	✗	Fair	(72)	http://www.nhm.ac.uk/our-science/data/hostplants/

PlantGDB	MySQL-PHP-Perl-Apache**TableMaker:** for query and retrieval	✓ (via ftp, FASTA format, GenBank, GFF3 or EMBL format, bzip2 files and MySQL tables)	✓ (Users can submit annotations)	✓ (Genome browser)	✓ (Sequence ID, Sequence Search)	✗	✓	✓ (**BLAST, BioExtract Server, DAS, FindPrimers, GeneSeqer, GenomeThreader, MuSeqBox, PatternSearch, ProbeMatch, TE_Nest, Tracembler, yrGATE**)	✓	✓	Fair	(73)	http://www.plantgdb.org/Website is no longer updated (01/07/2015)
						23/07/2012							

Phytozome	LAMPJ stack (Linux, Apache, MySQL, PHP/Perl and Java)	✓ (via the JGI Genome Portal after log in- OBO format, HTML table and tab delimited text)	✓	✓ (Gbrowse)	✓ (Keyword Search)	✓	✗	✓ (JBrowse, BLAST, BLAT, PhytoMine, BioMart)	✓	✓	Fair	(74)	http://www.phytozome.net/ (DB is well developed and being updated as Versions 9.1, 10.0,10.1, 10.2, 10.3, 11 etc.)
						23/02/2016							

PLAZA	NA	✓ (via ftp-directory-different formats-.csv, .tfa),	✗	✓ (AnnoJ and Genome View)	✓ (Multi-optional search)	✓	✗	✓ (BLAST and various tools to explore/identify gene families, etc.)	✓	✓	Fair	(75, 76)	http://bioinformatics.psb.ugent.be/plaza/ (DB is well developed and being updated as Versions 1, 2, 2.5, 3.0 etc.)
						26/06/2015							

ChromDB	MySQL, HTML, Mason, (Perl, HTML), UNIX	✗	✗	✓ GBrowse	✓ (Quick Search & Advanced Search)	✓	✗	✓ (BLAST, GMOD, GBrowse)	✓	✗	Fair	(77)	http://www.chromdb.org/index.html (Publication is available but link is not functional)

PlantTFDB	MySQL	✓ (FASTA, etc.)	✗	✓ (Browse by Species, families)	✓ (Quick Search & Advanced Search)	✗	✓	✓ (BLAST)	✓	✗	Fair	(78–80)	http://planttfdb.cbi.pku.edu.cn/ (PTFDB v1.0,2.0 and 3.0 available)
						23/08/2013							
PLANTS Database	NA	✓ (uncompressed ASCII text)	✗	✓	✓ (Name Search, State Search, Advanced Search)	✓	✗	✓ (Crop Nutrient Tool, etc.)	✓	✗	Fair	(81)	http://plants.usda.gov
						19/10/2015							

Pest and pathogen databases													

SilkPathDB	NA	✓ (FileServer- PNG, SVG, FASTA, GFF,	✓	✓ (Browse GO)	✓ (Keywords, sequence IDs, Locations)	✓	✓	✓ (WEGO, BLAST, EuSecPred, ProSecPred)	✓	✗	Fair	–	http://silkpathdb.swu.edu.cn/
						08/07/2015							

Combined databases													

Barcode database													

BOLD	PostgreSql, Java, C ++, PHP, Linux system	✓ (**Specimen data:** XML or TSV, **sequences:** FASTA **Trace files:** .ab1 or .scf, **Both specimen details & sequences:** XML or TSV)	✓(trace files from ABI sequencers)	✓ (Taxonomy Browser)	✓ (Keyword search)	✓	✓	✓ (Distribution Map Analysis, Taxon ID Tree, Distance Summary, sequence composition tools etc.)	✓	✓	Fair	(86)	www.barcodinglife.org

Taxonomy/distribution related databases													

DBIF	NA	✗	✗	✓	✓ 3 options -(‘Search for …’-invertebrates, host plant, source)	✗	✗	✗	✓	✗	Fair	No publication for the DB but related publications are available	http://www.brc.ac.uk/dbif/

DBMW	NA	✓ (from Aim & Scope Page)	✓	✓ (catalogue)	✓ (catalogue, biblio-graphy, images)	✗	✓	✗	✓	✗	Fair	No Publication	http://www.nhm.ac.uk/research-curation/research/projects/butmoth/Introduction.html
						06/2013							

CNIDB	NA	✓ (pdf)	✓	✓	✓ (Common Names of Insects and Related Organisms)	✓	✗	✗	✗	✓	Fair	No publication	http://www.entsoc.org/pubs/common_names
						02/03/2016							
BAMONA	NA	✓ (Images are available)	✓	✓ (Browse All Species)	✓ (Search for Species Profiles-Under Image gallery section)	✓	✗	✓ (Identification Tools)	✓	✓	Fair	No publication	http://www.butterfliesandmoths.org/
						08/03/2016							

EPPO Global DB	NA	✓ (Documents-pdf, Image-jpg)	✓	✓	✓ (Advanced Search)	✓	✗	✓ (Fast text and Batch processing tools)	✓	✓	Fair	EPPO Global Database (2015)	https://gd.eppo.int
						01/2016							

EOL	NA	✓	✓	✓	✓ (EOL Search)	✓	✗	✗	✓	✓	Fair	(122)	http://www.eol.org
						23/02/2016							

ITIS	MySQL, PostgreSql	✓ (ITIS Downloads-Informix 7, MS SQL Server, MySQL bulk load, MySQL by table, PostgreSql, SQLite).csv format	✓	✓	✓ (Quick Search, advanced search)	✓	✗	✓ (Compare Taxonomy/Nomenclature, The Taxonomic Workbench tool)	✓ (Guidelines provided)	✗	Fair	No publication	http://www.itis.gov/
						01/03/2016							

INPN	NA	✓	✓	✓	✓ (Search data on program, species & habitat)	✓	✗	✓ (Data & Tools option)	✗	✓	Fair	No publication	http://inpn.mnhn.fr/
						09/03/2016							

Pheromone database													

Pherobase	NA	✗	✓	✓ (Browse data in various categories)	✓ (Search Bar)	✓	✓	✓ (Kovats Calculator, Formula Generator)	✓	✓	Fair	(88)	http://www.pherobase.com/

Silk databases													

Biomat_dBase	PHP, HTML/CSS, Javascript, Red Hat Enterprise Linux 4	–	–	–	–	–	–	–	–	–	Poor	(89)	http://dbbiomat.iitkgp.ernet.in/(But Link is not functional)

SRDB	Oracle-MySQL-PHP associated with technologies like GIS, RS, Flex and C#	–	–	–	–	–	–	–	–	–	Poor	(90)	No link available
SFSDB	SQL server 2000, Visual Basic.NET	–	–	–	–	–	–	–	–	–	Poor	(91, 92)	No link available

Other web resources													

miRNEST v2.0	HTML, CSS, PHP 5.2.11 and MySQL 4.0.31	✓(.gz)	✓	✓	✓	✓(version 2.0 is currently available)	✓	✓ (HuntMi)	✗	✗	Fair	(93, 94)	Integrated database available at http://lemur.amu.edu.pl/share/php/mirnest_1.0/home.php and http://mirnest.amu.edu.pl

miRBase	MySQL	✓ (via ftp)File formats-FASTA sequences, GFF genome coordinates and MySQL database dumps	✓	✓ (Browse miRBase by species)	✓ (Search miRBase)	✓	✓	✓ (BLASTN, SSEARCH)	✓	✗	Fair	(95–98)	http://www.mirbase.org/ miRBase 21 is currently available (released on 26/06/2014)
						03/07/2014							

MEROPS	MySQL, Distributed annotation system (DAS) server	✓(After log in)	✓	✓	✓ (Searches)	✓	✓	✓ (BLAST)	✓ (in the form of PPT)	✓	Fair	(99, 100)	http://merops.sanger.ac.ukVersion 9.13 is currently available (released on 06/07/2015)
						06/07/2015							

For the ease of writing the manuscript, abbreviations of the databases were used within the text. Of all the abbreviations, some were predefined by the database creators while some were defined by authors of this manuscript.

## Silkworm databases

Silkworms form the backbone of seri-ecosystem and extensive research has been done on it. Currently, there are about 20 databases available which comprise of silkworm specific information (Figure 3). According to the data type, these databases can be broadly categorized as nucleotide (13 numbers), protein (04 numbers), genetic resource (02 numbers) and pathway (01 number) databases which are briefly described and compared here.

### Nucleotide databases

Nucleotide databases constitute diverse nucleotide information like genomic sequences; expressed sequence tags (ESTs), microarray, microsatellites, transcriptomic data, etc. They provide fast and easy accessibility of sequence information for biological, functional, comparative genomics and phylogenetic studies. The available nucleotide databases are briefly described as below.

#### Silkworm genome databases

The first draft genome of the lepidopteran model organism, *B. mori* was published in 2004 with 3× and 6× coverage by two independent research groups from Japan and China respectively (17, 18). In the same year, as an outcome of this research, ‘SilkDB’ had been published from China, containing 6× draft genome sequence and integrated information on chromosomal maps, cDNAs, ESTs, transposable elements (TEs), annotation, orthologous groups in the form of genes, etc. (19). ‘SilkDB’ also known as ‘Silkworm Knowledgebase’ is the first comprehensive genomic database of *B. mori* that has been developed by *Beijing Genomics Institute (BGI), China*. The entire data in SilkDB has been organized into three modules: (i) scaffold, (ii) gene and (iii) TE linked together by the MapView program which can be accessed through keyword or BLAST search (Supplementary Table S1). The scaffold module comprises of 23 156 scaffolds for 28 chromosomes; the gene module consists of 18 510 annotated gene sequences and full cDNA sequences of 212 known silkworm genes; and the TE module hosts around 601 225 TEs (19). This database was designed and implemented in *Oracle9i relational database management system (RDBMS)* using *JSP scripts* under *TomCat web server* and accessible at http://silkworm.big.ac.cn/index.jsp.

The 3× and 6× genomes carried insufficient genome sequence data due to low coverage as compared to that of other species like *Drosophila melanogaster* and *Anopheles gambiae* (20, 21). Therefore, after 3 years of publication of the draft genome, both these datasets were merged and reassembled by the ‘International Silkworm Genome Sequencing Consortium’ (ISGSC, 2007) to generate the same genome (432 Mb) with a remarkable coverage of 8.5× (22). In order to accommodate the new integrated and comprehensive genomic information, ‘KAIKObase’ and ‘SilkDB v2.0’ (upgraded version of SilkDB) were published in 2009 and 2010 respectively (23, 24). KAIKObase was developed by *National Institute of Agrobiological Sciences (NIAS), Japan* under Silkworm Genome Research Program (SGRP); while SilkDB v2.0 was developed at *Institute of Sericulture and Systems Biology, Southwest University (SWU), China.* KAIKObase harbors genome data for functional studies, BAC-end sequences, fosmid sequences, physical/genetic maps and EST sequences; while SilkDB v2.0 includes whole genome assembly, gene annotation, chromosome mapping, microarray expression, ESTs, etc. (Supplementary Table S1). KAIKObase was constructed using *PostgreSQL version 8.2.1* and implemented in *Javascript* (23). The current version of ‘KAIKObase v3.2.2’ consists of four map browsers (PGMap, UnifiedMap, GBrowse, UTGB), one gene viewer (GeneViewer) and five independent databases (Bombyx Trap Database, KAIKO2DDB, KAIKOGAAS, full length cDNA database and EST database). The database can be accessed through advanced three-way data mining approach-‘Chromosome Overview’, ‘Keyword and Position search’ and ‘Scaffold Sequence Search’. KAIKObase is accessible at http://sgp.dna.affrc.go.jp/KAIKObase/. On the other hand, SilkDB v2.0 is equipped with several user-friendly tools like Genome Browser, WEGO, ClustalW, CAP3, SilkMap, etc. in addition to BLAST (24). This version is implemented in *MySQL* (http://www.mysql.org/) *database management system* and navigated by *GBrowse* similar to KAIKObase. The database can be accessed at http://www.silkdb.org. Both KAIKObase v3.2.2 and SilkDB v2.0 are well developed and user-friendly databases with various inbuilt analysis tools. Users can upload and download map specific data from GBrowse option in KAIKObase v3.2.2. It is more advantageous for the users to use SilkDB v2.0 because it has a dedicated download page linked to an ftp server, while the download process in KAIKObase is quite complicated (Table 1). Additionally, both of these databases (KAIKObase was last updated in 2013 and SilkDBv2.0 in 2009) can be updated for their better usability. Although well-developed genome databases of *B. mori* are available as discussed above, there is a scarcity of genomic data related to other silkworms, particularly the wild silkworms. This may be attributed to the issues related to their domestication issue due to which they are still unexplored. Genome information is necessary for various downstream studies like mutation, mapping studies, etc. Development of new databases or integration of such data in the available databases will help in the analysis of their genome function and evolution.

#### *Silkworm* *gene* *expression* *databases*

Studies on genes using approaches like microarrays, NGS, etc. can help us in understanding gene expression and regulation under variant conditions. ESTs, microarrays and transcriptome aid in functional genomics by providing information required for genome annotation, detection of aberrant transcription, high-throughput (HT) genotyping of large populations, tissue specificity, pathogen infection-dependent gene expression, sex specificity, etc. (25–28). Gene expression studies have been applied on *B. mori* and other silkworms, leading to the development of databases which include three EST databases (‘SilkBase’, ‘WildSilkbase’ and ‘ButterflyBase’), one microarray database—‘*Bombyx mori* Microarray Database’ (BmMDB) and one transcriptome database (‘SilkTransDB’).

Among the three EST databases, SilkBase hosts EST sequences of five lepidopteran insects (*B. mori, B. mandarina*, *S. cynthia, Ernolatia moore* and *Triloca varians)*, WildSilkbase hosts EST data of three economically important silkmoths (*A. assamensis*, *A. mylitta* and *S. cynthia*) of Saturniidae family and ButterflyBase integrates the EST sequences as well as their annotation data of butterflies and other lepidopteran insects (Supplementary Table S1). SilkBase was constructed via collaboration among various institutes like *NIAS Japan, University of Tokyo*, etc. and published in 2003 (29). WildSilkbase and ButterflyBase were developed by *Centre for DNA Fingerprinting and Diagnostics (CDFD), India* and *Max Planck Institute for Chemical Ecology, Germany* and published in 2008 (30, 31). The EST data of all three databases were derived via sequencing of cDNA libraries. SilkBase comprises 35 000 ESTs obtained from 36 cDNA libraries while WildSilkbase contains 57 113 ESTs generated from 14 cDNA libraries. Both databases possess datasets extracted from different tissues and developmental stages of silkmoths. ButterflyBase, being a secondary database, hosts information on 273 077 ESTs obtained from primary sequence databases like EMBL/GenBank/DDBJ database for 30 diverse species and their protein translations (31). A unique feature of SilkBase is that it has cross-referenced data ensuring reliability of its data. WildSilkbase operates with a *MySQL-PHP* based interface on an *Apache web server* whereas ButterflyBase uses *PostgreSQL* with a customized version of the PartiGene schema (a tool used for developing partial genomes) (30–32). Database and web interface development data on SilkBase could not be discussed due to lack of information in its related publication. Other technical aspects of WildSilkbase and SilkBase like search visibility, data download, analytical tools, etc. are comparably fair. Inbuilt search options of SilkBase include all variations of BLAST, search with variable options like keywords, gene model, genome position, EST, etc. for three organisms (*B. mori, B. mandarina* and *S. cynthia ricini*) as well as browsing options available under ‘Libraries’ tab. Search options in WildSilkbase are slightly similar to SilkBase with options like ‘keyword search’, ‘Homology Finder’, ‘SSR Finder’ (Simple Sequence Repeat Finder) as well as inbuilt BLAST tool (blastn, tblastn and tblastx). However, there are limited options for search in ButterflyBase (simple text queries for pre-computed BLAST results). Other analytical tools in at least one of the three databases include ‘GO (Gene Ontology) Viewer’, ‘cSNP’ (SNP prediction tool), etc. Two features unique to ButterflyBase are the presence of a protein prediction tool, prot4EST and a scheme to provide services like EST and mRNA data processing prior to submission to dbEST of NCBI. This feature is absent in the other two databases. However, ButterflyBase developers should address the issues with its basic accessibility and perform search engine optimization to increase its web visibility (Table 1).

Till now, SilkBase (*Bombyx* EST Database) has the most number of citations (∼225) for its related publication among the three EST databases as well as other silkworm databases (Supplementary Table S2). Some features that account for its widespread popularity are the spectrum of search options discussed above, presence of numerous analytical tools, ease of navigation, etc. Also, SilkBase was the first database reported exclusively for a silkworm, so its early implementation has a role to play in its popularity. ‘SilkBase’, ‘WildSilkbase’ and ‘ButterflyBase’ can be accessed at http://silkbase.ab.a.u-tokyo.ac.jp/cgi-bin/index.cgi, http://www.cdfd.org.in/wildsilkbase/home.php and http://www.butterflybase.org/ (currently not functional) respectively.

Other gene expression data available on silkworms are microarray data from ‘BmMDB’ and transcriptome data from “SilkTransDB”. “BmMDB” is the first and only microarray database of *B. mori* developed by the *Institute of Sericulture and Systems Biology (ISSB), Southwest University, China* (33). It offers tissue-specific gene expression profile obtained through genome-wide (22 987 no. of 70-mer oligonucleotides) microarray analyses in a fifth instar silkworm (Supplementary Table S1). Published in 2007, it is the second most cited database (∼193) next to SilkBase among silkworm databases (Supplementary Table S2). It is integrated with SilkDB and contains a BmArray-map to display raw data. BmMDB is based on a *PHP-HTML* web interface with its back end linked to a *MySQL* database and can be searched with sequence (BLAST) and text-based (Probe ID) search options. The search output includes functional annotation of genes (name and CDS), experimental raw data, etc. Data submission and ftp download options are absent in BmMDB (Table 1).

The fifth gene expression database, ‘SilkTransDB’, was developed by *Chinese Academy of Agricultural Sciences, China* to integrate transcriptome and genome annotated data from SilkDB (34). It comprises of whole transcriptome information of different developmental stages of *B. mori* obtained through HT RNA sequencing (RNA-Seq). This data has expanded the information on silkworm genome by identifying ∼5500 novel transcripts and 13 195 new exons, thus uncovering the functional complexity of *B. mori* transcriptome (Supplementary Table S1). The core data of SilkTransDB consists of 3.3 gigabase (Gb) reads covering around 7-fold of *B. mori* genome and protein-coding genes that constitute 81.3% of all the predicted genes in SilkDB (34). SilkTransDB can be browsed through *GBrowse* and searched using multiple BLAST options (blastn, tblastn and tblastx). According to the publication, it has three web-interfaces: (i) SilkDB annotation (gene, CDS and mRNA), (ii) transcriptome information (gene, structure and alternative splicing (AS) events) and (iii) Map-solexa data of reads and coverage. However, this description does not comply with the interface displayed over their respective website. SilkTransDB accepts submission of annotated data files using GBrowse.

The status of data update in BmMDB and SilkTransDB is unclear due to the absence of relevant information in their websites. Also, there are accessibility issues associated with ‘BmMDB’ website (http://www.silkdb.org/microarray/) which should be addressed for making it usable. ‘SilkTransDB’ can be addressed at http://124.17.27.136/gbrowse2/.

The gene expression data, viz. EST, microarray and transcriptome, from the databases discussed above, will help in the identification of unique lepidoptera-specific ESTs, unique genes and proteins as well as development of molecular markers and identification/annotation of unknown proteins. These data can also have implications on evolutionary studies on insects. Apart from the techniques used above, gene expression data can also be generated via Serial analysis of gene expression (SAGE), Fluorescence *in situ* hybridization (FISH), etc. which can be included in these databases to broaden their data spectrum, or exclusive databases can be developed for the data derived from these techniques alone (35, 36).

#### *Microsatellite* *databases*

Microsatellites are the repeated sequences of 1–6 bp length which are widely used in fingerprinting, linkage analysis, marker studies, etc. (37). ‘SilkSatDb’ was the first microsatellite database of silkworm *B. mori* created by *Centre for DNA Fingerprinting and Diagnostics (CDFD), Hyderabad, India* in 2005 (38). It consists of microsatellite data derived using SSRF program from whole genome shotgun (WGS) and EST sequences of *B. mori* (39). It also contains data on mutations and polymorphisms, allelic frequencies, evolutionary conservation of microsatellites, etc. (Supplementary Table S1). In addition, it catalogues extraction protocols, validated primer sequences for around 200 loci (under the tab ‘PrimerBase’), informative figures and methodologies adopted for inter simple sequence repeats (ISSRs)-based genotypic analyses. A database with similar type of data but with wider range of organisms was constructed by the same laboratory in 2007 (40). Named ‘InSatDb’, this database comprises of microsatellite information from five completely sequenced insect genomes (*B. mori, D. melanogaster*, *Apis mellifera*, *Tribolium castaneum* and *Anopheles gambiae*) derived using a different tool Tandem Repeat Finder version 4 (40, 41). Various characteristics of microsatellites like nature, type, frequency, motif, genome location, repeat size, copy number, etc. of the five insects can be retrieved from this database (Supplementary Table S1).

Both are *MySQL* relational databases developed using *PHP* as a server side scripting language. The search page interface is similar in both the databases; the output interface for InSatDb is interactive, displaying an array of information like ‘Repeat Kind’, ‘Start’, ‘End’, ‘Copy Number’, ‘Length’, etc. and is linked to a primer-designing tool ‘Primer3’ while that of SilkSatDb is linked to two analytical tools ‘AutoPrimer’ and ‘SSR Finder’. As of 11 March 2016, the search engine was dysfunctional due to ‘database connection’ issues. ‘InSatDb’ is more advantageous to use over ‘SilkSatDb’ due to its wide scope of comparative genome analysis among five insects, batch download options and a ‘tutorial’ link; in fact, the data of SilkSatDb can be found within InSatDb with additional information. Both databases lack periodical updates and data submission features (Table 1). ‘SilkSatDb’ is accessible at http://www.cdfd.org.in/SILKSAT/index.php and ‘InSatDb’ at www.cdfd.org.in/insatdb. Similar to the above two sections, the microsatellite databases are available only for *B. mori* while being unavailable for other lepidopteran insects, particularly wild silk moths. Genomic information can greatly assist microsatellite studies as discussed above and thus, opens new scope for constructing more microsatellite databases.

#### *Silkworm* *mutant* *databases*

Being a model organism for insects, especially silkworms, *B. mori* mutants are generated for research as well as commercial interests and are expected to fulfill requirements like enhanced production of fibroin, different colored silks, etc. (42). Web search currently shows three databases that host information on such mutants, namely, ‘Database of *Bombyx* mutant photographs (DBMP)’, ‘ABURAKO Database’ and ‘Bombyx Trap database’. ‘DBMP’ has been developed by *Laboratory of Insect Genetics and Bioscience, University of Tokyo, Japan* to provide photographs of the mutants having mutation on different chromosome numbers. Also, this database is integrated with full-length cDNA database and SilkDB. The other two databases, i.e. ‘ABURAKO’ and ‘Bombyx Trap database’ have been developed and maintained by *NIAS, Japan.* ‘ABURAKO’ consists of information (20 mutation loci, alleles, list of mutants, translucence level, amount of uric acid accumulation and other characteristics) on silkworm mutants with translucent larval skin due to deficit of uric acid metabolism, including low-resolution images for some of the mutants. In ‘Bombyx Trap database’, reporter expressions in 288 transposon insertion lines (enhancer and gene trap lines) via transposon mutagenesis are included (23, 42). Also, the information regarding the position of mutation in a genome sequence, fluorescent intensity of reporter expression at various developmental stages, reporter type, etc. are available in this database. It has text-based and image-based search options. The text-based search provides information on strain ID, reporter used, measured fluorescence site, etc. while the latter is a browsable gallery of good-resolution images for normal and fluorescent eggs, larvae, moths, etc. Both ABURAKO and DBMP lack an in-built search engine.

A critical comparison of these three databases on silkworms mutants with the other databases discussed above makes it apparent that they require enormous improvement in various matters (Supplementary Table S1). The web interface of all the three databases is not well-designed; only one has a search engine; none of them contain data submission or download links and none of them are updated periodically (Table 1). Also, to which extent can an image help a researcher is a questionable issue. Overall, if these three databases can be unified to create a common database with the addition of missing features described above, then the resultant database will have a better application periphery. Another meaningful addition to this group can be the creation of a database of mutant generation protocols used by researchers. One can access ‘DBMP’, ‘ABURAKO’ and ‘Bombyx Trap database’ at http://papilio.ab.a.u-tokyo.ac.jp/genome/, http://cse.nias.affrc.go.jp/natuo/en/aburako_top_en.htm and http://sgp.dna.affrc.go.jp/ETDB/, respectively.

#### *Transposable elements (TEs)* *databases*

‘BmTEdb’ is the only database exclusively available for transposable elements of *B. mori* hosted by *Chongqing University, China* (43). It is a comprehensive database on 1308 TE families which have been further classified into sub-families. TEs are said to represent ∼40% of the silkworm genome (44). The researchers have used a combined (*de novo*, structure-based and homology-based) approach to identify and classify the TEs within the *B. mori* genome (43). TEs play a role in the function and evolution of genes/genomes which makes the database useful for researchers trying to understand the role of these mobile elements in silkworm genetics (45, 46). BmTEdb provides users with options to search, browse and download the TE sequences in single as well as in batch. Addition of analytical tools like BLAST, HMMER and GetORF enhances the analytical scope of this database (47–49). Options like public data submission (suggestions available) and update, user account sign in, etc. are not available within the database (Table 1). BmTEdb can be accessed at http://gene.cqu.edu.cn/BmTEdb/.

Study of transposons including their identification, characterization and annotation, is crucial as it provides insights into genome variation and evolution. This can be greatly facilitated by genomics, genetics, transgenic technologies, HT sequencing technologies, etc. In addition to *B. mori* which has been studied well in BmTEdb, there is a great scope of developing databases for many other related silkworms.

#### Other web-resources

Apart from the above databases, the sequence and frequency information of the ovarian small RNAs in *B. mori* can be retrieved from a web-platform ‘Silkworm sRNA’ supported by National Bioresource Project (http://www.nbrp.jp/). Currently, it contains a total of 67 700 counts of RNA of 38 493 kinds which are available at http://papilio.ab.a.u-tokyo.ac.jp/small_RNA/all_smallRNA.txt.

### Protein databases

The interest in studying silkworms is deeply rooted in the proteins (fibroin and Sericin) that it produces. Therefore, protein databases serve as an essential platform in studying gene expression, post-translational modifications and other biological processes related to silkworm proteins (50). Till now, four databases are available directly related to this area, namely, ‘KAIKO2DDB’, ‘SilkProt’, ‘SilkPPI’ and ‘SilkTF’.

‘KAIKO2DDB’ (Silkworm proteome database or SPD) was the first silkworm proteome database published by *NIAS, Japan* in 2006 (23, 50). It houses the 2D gel-electrophoresis and mass spectrometry information of seven major tissues of silkworm (midgut, malpighian tubule, ovary, middle silk gland, posterior silk gland, fat bodies and hemolymph) (Supplementary Table S1). The data can be accessed by accession number, description ID or gene name, author, spot id/serial number, identification methods and pI/Mw range. The database was developed using *Make-2DDB II* software and is hosted on a web interface based on *HTML*. The other three databases: ‘SilkProt’, ‘SilkPPI’ and ‘SilkTF’ were developed by *Bioinformatics Centre, CSR&TI (Central Sericulture Research and Training Institute), Mysore, India.* SilkProt database contains annotated protein data of silkworm which helps in predicting structure and pathways. SilkPPI, i.e. Silkworm Protein–Protein Interaction database provides details on protein–protein interactions of *B. mori* which facilitates the study of biological and cellular processes (51, 52). It uses protein sequences from SilkDB along with computational methods, e.g. interlog based method for data predictions (24, 51). Around 7736 protein interaction pairs including 2700 unique proteins that were predicted using Interlog method are included in the database (51). SilkDB accession number can be used to search the database for the information regarding interaction proteins, GO annotation, Pfam domains and nominal *P*-value of the microarray data. The database can be accessed through http://210.212.197.30/SilkPPI/ but currently the link is non-functional (11 March 2016). Again, Silkworm transcription factor (SilkTF) database hosts information on transcription factors (TFs) of *B. mori* silkworm. The database can be browsed and searched either by SilkDB sequence ID or domain search. ‘Sequence search’ facilitates finding of transcription factors present in the sequence, PfamID, domain name, regions and e-value information; ‘Domain search’ tool gives an output of list of sequence IDs having specific domain, locations of sequences with the specific domains and their corresponding e-values.

Among the four databases, KAIKO2DDB is developed slightly better than SilkProt, SilkTF and SilkPPI. The latter two have issues related to accessibility. SilkProt can have a better web interface rather than having the search engine as its homepage. Due to the lack of home page, it does not offer users any other options like data upload/download, data analysis or help page. SilkTF has similar problems to that of SilkPPI, except that it has an in-built BLAST tool (Table 1). KAIKO2DDB has numerous search options and global search options (50). It can be accessed through KAIKO Proteome Database (http://KAIKO2DDB.dna.affrc.go.jp/) or SWISS-2DPAGE (http://kr.expasy.org/ch2d/make2ddb/) under the silkworm genome database. SilkProt, SilkTF and SilkPPI are accessible at http://www.btismysore.in/silkprot/, http://www.btismysore.in/SilkTF/ and http://210.212.197.30/SilkPPI/, respectively. Apart from these, an upcoming database ‘Silkgpcr’ has also been reported on the web page of *Bioinformatics Centre, CSR&TI, Mysore, India.* It will aim to provide information about the G protein coupled receptor protein and its various classifications (Rhodopsin like, Secretin like receptor, Metabotropic glutamate receptors, etc.) in *B. mori.*

Unlike nucleotide databases, there is dearth of databases related to the protein structure, sequence, protein–protein interactions, etc. The four databases discussed above have a common data type protein, however, they address three different facets. Similarly, new databases focused on silkworm protein structure or sequences can be developed by the researchers. Implementation of combinatorial approach (Proteomics and Transcriptomics) can be one of the ways to understand how variation in the proteome and transcriptome is associated with physiological changes in silk production, to characterize strains, etc. The scope of database development in this field is huge, so the issues of silk protein data scarcity should be addressed.

### Silkworm genetic resource databases

Silkworm genetic resource databases which deal with data like varieties, strains, races, etc. other than genomes, transcriptomes and genes, also form an integral part of seri-databases. ‘Silkworm Gene Resources database’ (SGRDB) and ‘SilkwormBase’ are the two databases that can be classified under this group. SGRDB is a *MySQL* relational database developed by *ERWin Data Modeler* software where the data is stored in *Oracle relational database management system* and maintained by *National Academy of Agricultural Science (NAAS), Korea*. SilkwormBase is an integrated genetic resource database of silkworms developed as a part of *National BioResource Project (NBRP)* between the resource centre (Graduate School of Agriculture, Kyushu University) and the information centre (National Institute for Genetics). SGRDB provides the characterization information (e.g. strain, accession number, color, shape, etc.) of 321 varieties collected from different regions including Korea, China, Japan, Europe, tropical region and non-classified group along with 1132 photo images of different life stages of these silkworm varieties. It also allows the users to access information regarding silkworm races such as univoltine, bivoltine, multivoltine and others (53). SilkwormBase hosts around 456 phenotypically classified strains and a total of 419 genes. It also facilitates the users to access information regarding genetic stock resources including strains, larval period, images of strains at different stages (egg, larva, pupa and adult), feeding habits of artificial diets, etc. and enlists the genes expressed at various life stages, their features, classification as well as linkage maps (Supplementary Table S1). SGRDB has four main functional categories, namely, variety search, characterization viewer, photo gallery and general information whereas SilkwormBase is equipped with three search options: ‘Strain’, ‘Gene’ and ‘References’ along with an additional option ‘Distribution request’ where one can online request the eggs or other developmental stages such as larva, pupa and adult of various silkworm strains. ‘SGRDB’ and ‘SilkwormBase’ are available at http://www.naas.go.kr/silkworm/english (not accessible on 11 March 2016) and http://www.shigen.nig.ac.jp/silkwormbase/about_kaiko.jsp, respectively.

SilkwormBase (both English and Japanese versions) is found to be more helpful in comparison to SGRDB since it is accessible and regularly updated (last updated 27 April 2015). It also links to a new website (last updated: 5 December 2014) created as a part of NBRP which deals with the collection, preservation and distribution of wild silkworms (*S. cynthia pryeri* Butler, *S. cynthia ricini* Donovan, *A. yamamai* Guérin-Méneville, *A. pernyi* Guérin-Méneville and *Rhodinia fugax* Butler). Since there are a diverse range of wild silkworms existing in the world, exploration and inclusion of those genetic resources can be a remarkable feature of this database. Alternatively, new genetic resource databases can be developed to fill up the gap in seri-related field.

### Insect pathway databases

‘iPathDB’ is the only insect pathway database that houses pathway data on Lepidoptera (10 different orders) with a total of ∼52 insects. It was developed by *Li Lab: Insect Genomic and Bioinformatics Lab, China* in 2014 (54). Currently, 12 111 pathways for 52 different species associated with disease, xenobiotic metabolism signaling, insect hormone and wing development are available in this database. iPathDB has options for search (drop-down schematic and text-based). Its strongest feature is the inclusion of a pathway construction software ‘iPathCons’ which facilitates the users to construct pathways from transcriptome as well as official gene sets (OGSs) data of insects. Users can download the pathways constructed through iPathCons by sorting species list or species on the phylogenetic tree. Additionally, it provides batch download for the raw data files and in-built softwares (Table 1). The database was designed using *HTML, PHP, CSS* and *JavaScript* which operates under *Apache HTTP server.* These pathways will be helpful for entomological research community and are available at URL: http://ento.njau.edu.cn/ipath/. iPathDB has tried to cover necessary pathways and can act as a better platform for various insect pathway studies. Further, it can act as a stand-alone database if it adds more pathways in a single platform. The pathways related to insect behavior, immunity, metabolism (carbohydrate and fatty acid synthesis/metabolism), function and evolution of genes and pathways involved in sex-determination, wounding/herbivory signaling pathways, etc. can be included to make it a full-fledged database or new databases can be constructed based on essential pathway studies.

## Silkworm host plant databases

Host plants are the most important resources in seri-ecosystem as they provide food and nutrition to the silkworms. Based on the preference of feeding by the silkworms, host plants can be divided as primary (1°), secondary (2°) and tertiary (3°) host plants. The quality and yield of silk produced by the silkworms depends on the selection of these host plants. For example, the cocoon color and tensile strength of cocoon fibers varies for polyphagous silkworms. Certain host plants of silkworms have economic importance other than sericulture and have been studied with different focus. For instance, fruits of *Morus alba* (mulberry) are a great source of nutrients and anti-oxidants (55). *Jatropha* is more popular as a biofuel crop and most of the molecular and genetic research has been focused on that aspect (56). Similarly, *Ricinus communis* is known for the production of castor oil having applications as lubricant, food, medicine, etc. (57). Those host plants, which are only specific for sericulture, have been rarely reported. Overall, the host plants can be further divided into domesticated and wild silkworm host plants. There are about 23 databases developed so far for these host plants which are discussed as below (Figure 3).

### Databases of mulberry

Mulberry is the primary host plant of *B. mori* belonging to family Moraceae. About 150 species of mulberry have been identified till date, which provide shelter to several sericigenous insects in nature. *B. mori* requires specific sugars, proteins and vitamins for its normal growth and silk gland nourishment. Mulberry leaves play a very important role in providing adequate amount of nutrients for the production of good quality cocoons (58). Recent advances in HT sequencing technology have led to the generation of several mulberry specific databases that are ‘Morus Genome Database’ (MorusDB), ‘Mulberry Microsatellite Database’ (MulSatDB) and other databases.

‘MorusDB’ was the first mulberry genome database constructed by *Southwest University (SWU), China* and recently published in 2014. This database, available at URL http://morus.swu.edu.cn/morusdb, houses a wide range of genomic and biological information of *M. notabilis* C.K. Schneid (Mulberry). The core data of MorusDB constitutes 236-fold coverage of 330.79 Mb assembled mulberry genome sequence and reference-based assembled transcriptome sequence (59, 60). This information includes annotated genes, GO, ESTs, TEs, orthologs and paralogs, horizontally transferred genes, taxonomy, etc. (Supplementary Table S1). It has a user-friendly web interface designed and implemented using *MySQL* and *PHP*, embedded with helpful analytical tools like BLAST, WEGO, GO browse, genome browser, etc. One of the main advantages is availability of data download feature provided by *FTP* and *File Browser* which allows specific and batch download of the genome and transcriptome data. However, MorusDB lacks some features found in other popular databases like GenBank such as public data submission, user-registration, etc. (Table 1). Addition of the former can widen the range and amount of data in it while that of the latter will make it easier to use for the public.

‘MulSatDB’, on the other hand, was the first mulberry microsatellite database constructed by *CSR&TI, Mysore, India*. It comprises of mulberry genome as well as EST based microsatellite (SSR) markers (61). Currently, it hosts 217 293 WG SSRs out of which 2772 SSRs were mapped to *F. vesca* chromosomes and 361 functionally annotated SSRs among 962 present EST SSRs. The markers can be searched and browsed through two search sections: ‘Whole genome’ and ‘EST’ based on various criteria’s (repeat size, repeat type, motif type, etc.). This database was based on *MySQL RDBMS* and its web interface was designed using *HTML, PHP* and *Javascript* operating on *Apache 2.2 web server*. The presence of various inbuilt analysis tools (CMap, Primer3plus and BLAST), public data query and submission makes it an interactive and user-friendly database. Unlike MorusDB, this database accepts data submission on new SSR markers, marker information, research projects and publications which should be further standardized. However, it lacks data download and help/FAQ features, addition of which will further improve the database utility (Table 1). ‘MulSatDB’ can be accessed at http://btismysore.in/mulsatdb/index.html.

Apart from MorusDB and MulSatDB, few other web-resources/databases on mulberry are under constructions which are hosted at the *Bioinformatics Centre, CSR&TI, India* (URL http://www.btismysore.in/pgene.html, http://www.btismysore.in/dbase.html). One is a relational database called ‘Mulberry Genome Database’ that provides data on molecular marker, DNA fingerprints, similarity and dissimilarity index matrices, phylogenetic relationship (in dendrograms) and marker segregation pattern. It is also available in the form of compact disks (CD). Other three databases include ‘Database of DNA sequences for important plant genes in mulberry’, ‘MulDis (A Comprehensive Mulberry Disease and Pest Database)’ and ‘Sample Web Application for Analysis of Molecular ID’. The first database is accessible via internet while the other two are not. ‘MulTF’ is another database proposed in their webpage for transcription factors of mulberry. While being informative, these resources lack proper representation as well as design of a database and are tough to access, browse or even understand as no help page or publication is associated with the resources. They require further refinement in different areas of which dynamic web design is prime importance. Improvement in the existing web resources and the development of planned ones would help in the future research of mulberry.

### Databases of castor

*Ricinus* *communis* (castor bean) has enormous economic and ecological importance as a popular biofuel crop (62). It also serves as the primary host plant of *S. cynthia* (Eri silkmoth). Much of the genomic and molecular studies on castor are completed focusing on its economic significance. This information can be helpful in understanding the silkworm and host plant interactions. Databases have therefore been developed and published on castor, however, few of these database URLs are currently not accessible. One such database is the ‘*Castor Database’* developed by *TNAU (Tamil Nadu Agricultural University), Coimbatore, India*. It hosts the phenotypic and germplasm data of the currently available castor varieties. About 294 different germplasm including 20 FC5 plants and YRCH (Yethapur *Ricinus communis* Hybrid) plants are documented here. Users can access the information on qualitative characters (such as type of internodes, spike shape, length of primary spike, compactness of inflorescence, branching pattern of the stem, petiole length, lacination of leaf, type of inflorescence) as well as the quantitative characters (number of lobes in leaves, height of the plant, nodes in main stem, etc.) of castor plants. In addition to these traits, the yield information of various germplasm is proposed to be included in this database. This will help the farmers to select the varieties of improved traits. However, the database cannot be currently accessed through its available URL: http://www.tnaugenomics.com/castor/index.php. It also suffers from demerits like lack of a search engine that can do specific searches, analytical tools, data submission or download options and a help/FAQ page (Table 1). A namesake of Castor database also exists at http://glbrc.bch.msu.edu/castor/login hosted and maintained by Michigan State University, but it is not an open source and is accessible to registered users with no option for new registration on the homepage.

Another database on castor is ‘JCVI Castor Bean Genome Database’ developed at *J. Craig Venter Institute*, *USA*, which hosts 4X draft assembled genome sequence of *R. communis* (∼400 Mbp) generated using whole genome shotgun strategy (63). The database also has ∼31 221 putative proteins and ∼50 000 ESTs generated from different tissues to aid in gene discovery and annotation. The data from whole genome assembly as well as auto-annotation is available to download from the ftp server. However, this database lacks a common browse or search page for the hosted data. Even GBrowse is non-functional (11 March 2016). The other analytical tool, i.e. BLAST (all categories) works fine with the data. One of its branches of data named as ‘Castor Bean TAs’ has been discontinued and users are referred to other related databases. This database is available at http://castorbean.jcvi.org/index.shtml.

‘CastorDB’ is a comprehensive knowledgebase DB for *R. communis* developed at *M. S. University of Baroda, India* (64). It was based on integration of the genome sequence information obtained from NCBI and the previously discussed JCVI Castor Bean Genome Database. The database facilitates the users to retrieve information on protein localization, domains, pathways, sumoylation sites, gene expression, protein–protein interactions, etc. Implemented using *MySQL, Perl API (application programming interface), Java, CGI, HTML* and *Javascript*, one of the important features of this database is the presence of three way search method- simple, advanced and similarity search based on BLAST tool. The database is accessible at http://castordb.msubiotech.ac.in. The URL has not been functional since Oct’ 2015 till date (11 March 2016) and hence, more information could not be provided on it.

### Databases of papaya

*Carica papaya* (Papaya) is a highly nutritious tropical fruit plant that is popular worldwide. It is also one of the important secondary host plants of *S. cynthia.* The 3× draft WGS of *C. papaya* Linnaeus was first published in 2008 with genome of size 372 Mb (65). It has facilitated the molecular and genetic study of the plant which has industrial and agricultural significance. Also, WGS and the largely sequenced sex-determining region of papaya have provided a deep insight into its genome structure and organization. The databases describing information on papaya include *Papaya-DB* and *CPR-DB* (Papaya Repeat Database).

‘Papaya-DB’ is an online genomic data resource of papaya which was developed by *Center for Applied Genetic Technologies, USA* and is accessible through URL: http://www.plantgenome.uga.edu/papaya/. It acts as an interface to various data including WGS, EST sequences, physical and genetic maps, sex-determining region, etc. It also offers access to the Plant Genome Duplication Database (PGDD) that enables users to perform whole-genome alignments with other plant species. While the database is linked to GBrowse for WGS data browsing, the links on the webpage are non-functional (11 March 2016). Being a sole representative of papaya genome database, it lacks many important features such as non-availability of data download/deposition options, analytical tools, help page, etc. (Table 1). Developers should plan to improvise the database to make it user friendly and easily accessible by incorporating the necessary features.

The other database, ‘CPR-DB’, developed at the *University of Maryland*, *USA*, provides data on repetitive elements of papaya which constitute ∼56% of its genome (66). These repetitive elements are TEs (52%), tandemly arrayed sequences (1.3%) and high copy number (HCN) genes (3%). Among transposons and tandem repeats (TRs), retrotransposons and microsatellites constitute the most abundant portion (about 43.3%), minisatellites (0.19%) and satellites representing the least portion of genome. However, the database does not have the typical web interface and is merely hosted via ftp server. The data (i.e. novel TE, TR sequences, HCN transcripts and protein sequences) can be downloaded as .fasta files but cannot be browsed or searched separately, which constitute the major demerits of CPR-DB (Table 1). The data is accessible at ftp://ftp.cbcb.umd.edu/pub/data/CPR-DB.

### Databases of *Jatropha*

*Jatropha curcas* is an economically important plant which has enormous potential for biodiesel production. It is also important for sericulture, being the secondary host plant of *S. cynthia*. Much research on *Jatropha* is available but at present, only one open-source database exists for this plant. ‘Jatropha Genome Database’, developed at *Kazusa DNA Research Institute, Japan*, hosts genomic information and DNA markers of *Jatropha* (67). Currently, the database consists of total 297 661 187 bp sequence elements with an average G + C content of 33.7%. Also, the presence of keyword search, data download via ftp server and constant updates are the strong suites of this database (Table 1). It has undergone many revisions (current version 4.5) and is highly cited (Supplementary Table S2). Homology based search option (BLAST) is also available as an analytical tool to search full length sequence, predicted CDS sequence, predicted amino acid sequence and unigene sequences. Additional features like help/FAQ and data submission will make the database more user-friendly. This database is available at http://www.kazusa.or.jp/jatropha/.

### Databases of cassava

Cassava is an important nutritious food, popular in the regions of Africa, Asia and South America (68). Besides *Papaya* and *Jatropha*, *Manihot esculenta* (Cassava) is also an important secondary host plant of *S. cynthia.* There are three databases available for cassava- *Cassava Genome Database* (CGDB), *Chinese Cassava Genome Database* (CCDB) and Cassavabase.

CGDB, developed at *Institute for Genome Sciences*, *USA* comprises of BAC-based fingerprint maps of an inbred cultivar of cassava and simultaneously provides data on cassava ESTs, assembled ESTs, WGS sequences and SNPs from physical maps as well as genes. It also aims to identify the traits associated with drought tolerance in cassava. On the other hand, CCDB, developed at *Fudan University, China*, provides BAC and cDNA libraries, annotated genome and transcriptome data on various pathways (gene discovery, starch metabolism, photosynthesis, drought/cold acclimatization, etc.), linkage maps, markers, etc. ‘Cassavabase’, another comprehensive database or rather a web portal, is different from the above two databases in matters of the information hosted by it. The database provides a combination of information ranging from genomic sequences to phenotypes, genetic maps, breeding programs, etc. This database has been developed for both researchers and breeders under *Next Generation Cassava (NEXTGEN Cassava) Breeding project* and hosted by *Boyce Thompson Institute for Plant Research, USA*. It employs the advanced breeding machineries to improve cassava productivity and yield.

CGDB, CCDB and Cassavabase are accessible through http://cassava.igs.umaryland.edu/cgi-bin/index.cgi, http://www.cassava-genome.cn and http://www.cassavabase.org/, respectively. All of them have GBrowse and download options. However, link for GBrowse tool in CGDB is not functional (as on 11 March 2016). BLAST links (blastn, blastp, blastx, tblastn and tblastx) for CGDB have selected databases for checking similar sequences with several matrices to use. Addition of features like search, data submission, database update, help, user-registration, cross-referencing of the hosted data, etc. will increase the reliability of both CGDB and CCDB databases. Cassavabase is an ideal database that can be used a model by other database developers. Some of its merits are user-friendly interface, incorporation of data analysis tools for breeders (breeder home, phenotype analyze, barcode tools, genomic selection and population structure) and researchers (BLAST, ontology browser), the genomic map of Cassava with markers, etc. It also has features like data submission, query search, help topics and manuals, etc. (Table 1).

Although the three cassava databases exist as web-resources, these have not yet published. Proper publication of these databases will help in detailed understanding of the databases.

### Databases of *Quercus*

*Quercus* or the oak is a popular timber tree and also the 1° host plant of oak tasar silkworm (*A. proylei*). ‘Quercus Portal’ is the first web resource which deals with almost all facets of *Quercus* data (69). It is an integrative web portal developed under the EvolTree project at *Institut National de la Recherche Agronomique (INRA), France* which provides information on genome, genetic resources, biodiversity, evolution, phylogeny and taxonomy of *Quercus* (tree/shrub)*.* Based on the information it carries, the portal is divided into eleven sub-databases. Among these, *Oak genome*, *EST* and *Candidate genes* databases are three genome/EST/gene related databases which comprise of whole genome sequence; three unigene sets for the genus *Quercus* (OCV1, OCV2 and OCV3); and putative genes related to biotic/abiotic stress, phenology and growth, respectively (70, 71). Others include marker and mapping information related databases which are *QuercusMap, CMap, SSR* and *SNP. QuercusMap* provides genotypic and phenotypic data on mapping pedigrees; *CMap* contains genetic and comparative maps while microsatellite and single nucleotide polymorphism data in candidate genes of oak are provided by *SSR* and SNP databases. Likewise, the phenotypic, genotypic, geographic, genetic diversity and fossil data of the oak trees and their populations can be accessed through *TreePop, (GD)^2^, Oak provenance* and *FossilMap* databases. Besides being highly resourceful, Quercus portal also has a global search bar which allows query search across all the above-mentioned databases. However, a useful additional navigation guide with help page will make the portal easily accessible (Table 1). Quercus portal, with all the resources mentioned above, is available at URL https://w3.pierroton.inra.fr/QuercusPortal/index.php?p=fmap.

### Other generalized plant databases

Apart from the host plant specific databases, several generalized databases exist which contain genomic, proteomic and taxonomic information of some silkworm host plants. These web-resources are non-specific and include data on the host plants which will further contribute to better understanding of their biology (Supplementary Table S1). One such database is ‘*HOSTS’*, a database of host plants of the lepidopteran insects (around 15%) around the world created at *Natural Museum of History, UK.* Since it consists of ∼180 000 records of host plants for about 22 000 Lepidopteran species from ∼1600 published and manuscript sources, HOSTS claims to be ‘the best and most comprehensive compilation of host plant data available’ (72). It has two good search modules: ‘Text Search’ or ‘Drill down search’ that allow the users to search information using two criteria’s: ‘Lepidoptera’ or ‘Host plant’. Search can be performed by family, genus, species names (only scientific names) and location to obtain the host plant data of respective insects. No record of update was found in HOSTS; regular updates, data download and addition of data submission option can broaden its knowledgebase. It is available at http://www.nhm.ac.uk/our-science/data/hostplants/.

Two general plant genome databases, *PlantGDB* and *Phytozome* are also available which were developed at *Indiana University* and *Department of Energy's Joint Genome Institute, USA*, respectively. Both contain genome data of many plant species including silkworm host plants (73, 74). PlantGDB contains ESTs, cDNA sequences and microarray probes while Phytozome (current version v11) hosts 55 annotated genomes clustered into gene families. The development of PlantGDB was done using *MySQL-PHP-Perl-Apache server* while that of Phytozome by *LAMPJ stack (Linux, Apache, MySQL, PHP/Perl and Java)*. Both databases have important features like data download, browse, help, search and analysis enabled via many embedded tools. PlantGDB provides multiple analytical tools (BLAST, BioExtract Server, DAS, FindPrimers, GeneSeqer, GenomeThreader, MuSeqBox, PatternSearch, ProbeMatch, TE_Nest, Tracembler, yrGATE); bulk data download facility via ftp server, and individual data files in several formats (FASTA, GenBank, GFF3 or EMBL format, bzip2 files and MySQL tables). Data download in Phytozome can be done through JGI Genome Portal only after user registration in OBO format, as HTML table and tab delimited text. Its analytical tools include JBrowse, BLAST, BLAT, PhytoMine and BioMart (Table 1). Both are highly cited comparative genomics databases (Supplementary Table S2) but PlantGDB has been discontinued in 1 July 2015 while Phytozome is regularly being updated with new versions. These two databases can be accessed at http://www.plantgdb.org/ and http://www.phytozome.net/, respectively.

Few other generalized databases include ‘PLAZA’, ‘The Chromatin Database’ (ChromDB), ‘Plant Transcription Factor Database’ (PlantTFDB) and ‘PLANTS’. PLAZA is a web-portal developed to perform comparative genomics and phylogenetic analyses (75, 76). The information of gene families and genome homology of important host plants e.g. *R. communis*, *M. esculenta*, *C. papaya* can be explored using ‘Analyse’ tools. The database is available through URL http://bioinformatics.psb.ugent.be/plaza/. *ChromDB* includes the sequence information of chromatin linked proteins of some silkworm host plants such as *R. communis*, *M. esculenta* *and C. papaya* (77). This database is available at http://www.chromdb.org/index.html. However, currently the link to access this database is not functional (11 March 2016). Another database, *PlantTFDB* provides the identification and classification data of TFs of few host plants of silkworms. Users can also download the list of TF families and protein sequences of TFs of the plants through the database link http://planttfdb.cbi.pku.edu.cn/ (78–80). Besides genomic and proteomic databases, taxonomic information also plays an essential role in studying the biology of a plant. *PLANTS* database is the one that includes data like images, classification, ecology, etc. of a few host plants (*M. esculenta*, *Quercus* spp., *Shorea robusta*) (81). The database can be accessed through http://plants.usda.gov/java/. All four databases are equipped with necessary features like search, help, analytical tools and download option (download lacking in ChromDB; Table 1).

Review of the host plant databases in this section showed that the number of specialized data resources is not enough and on an average, the ones that are available are not well-equipped. Most of them lack one or the other important feature. Lack of database development expertise may be one reason behind this. Merging information sciences with biological data has been going on for quite a long time now and it is time for plant scientists to update their set of skills. Another observation was the lack of cross-references and analytical tools in the databases, which should be made an obvious requisite for any biological database. It has also been observed that generalized host plant databases are more cited than the specific databases. For instance, Jatropha database (C-139) is cited next to Phytozome (C-637) and PlantTFDB v 2.0 (C-163) while the citation value of MorusDB, MulSatDB, etc. is small (Supplementary Table S2). Overall, host plant databases are fewer in number than silkworm or rather insect databases. There is a need to bring together the scattered data on these host plants together in one piece, as was done in the HOSTS database. Also, a need of plant-specific bioinformatics tools was seen which needs to be addressed sooner than ever, as the HT technologies are quickly generating vast arrays of data that needs to be scoured for meaningful outputs.

## Pest and pathogen databases

Silkworms in association with host plants inhabit diverse niches and get affected with several viruses, bacteria, fungi and parasites ranging from mutualistic symbiosis to pathogenesis. Study on these organisms is equally essential to understand the host–pathogen interactions, studying molecular mechanisms involved in the pathogenesis, host immune response, developing new strategies against infectious pathogens, etc. (82, 83). Keeping this in mind, ‘SilkPathDB’ was constructed as first pathogen database by *State Key Laboratory of Silkworm Genome Biology (SKLSGB)* at *Southwest University, China.* This database deals with genomic and biological data of a variety of silkworm pathogens including fungi, bacteria, virus and microsporidia. The data includes genome sequences, gene annotation, proteomic and transcriptomic profile of silkworms under infected conditions, etc. (84). SilkPathDB is a user-friendly and full-fledged database having all necessary features like search, browse, download, help and multi-analytical tools (SilkPathDB BLAST engine, SearchGO, Browse GO, Genome Browse, EuSecPred and ProSecPred). The database is constantly upgraded (last updated 08 July 2015) and users also have freedom to upload data in this database (Table 1). These features make this database highly useful to the users interested in lepidopteran and other insect-related pathogenetic studies. One can access the database at http://silkpathdb.swu.edu.cn/. Despite being fully developed, the database has not yet been published. Apart from SilkPathDB, a new comprehensive silkworm disease and pest database ‘SilkDis’, being developed by *Bioinformatics Centre* at *CSRTI, Mysore, India* has been mentioned at URL http://www.btismysore.in/dbase.html. It will aim to serve as a data resource on silkworm diseases and pests; providing detailed information on disease occurrence, infection mode, biotic/abiotic factors and effective pest/disease management approaches.

Silkworm–pathogen interaction studies suffer from a lack of understanding of silkworm pathology. It has been observed that among the seri-resources, pest and pathogen databases are the least in number. SilkPathDB can be expanded with more information on other pests and pathogens infecting various silkworms and their host plants. Moreover, new databases including pest and pathogens should be made in order to further explore the cross-talk among host, pest and pathogens.

## Combined databases

In addition to the silkworm, host plant and pest/pathogen databases, we have reviewed few other databases that comprise of generalized information of seri-resources (Figure 3). These databases are not only specific to any individual silkworm, plant or pest databases but contain integrated information of these resources. Seventeen databases have been found which are classified based on the data type (Supplementary, Table S1) and briefly described as below.

### Barcode databases

Barcodes are short DNA sequences serving as signatures for the identification and classification of species, process called as DNA barcoding (85). ‘BOLD’ (The Barcode of Life Data System) is one such user-friendly data resource developed by *Consortium for the Barcode of Life (CBOL)* to enable collection, storage, analysis and publication of DNA barcode sequences by amassing distributional, morphological and molecular information (86). Around 1 180 314 specimen records of lepidopteran insects including 53 476, 34 950 and 2429 members of Saturniidae, Sphingidae and Bombycidae families, respectively, are provided by BOLD (11 March 2016). Also, plants and fungi specimen records are available in this database. The data can be accessed through four main sections: (i) public data portal, (ii) a database of barcode clusters, (iii) a data collection workbench and (iv) an educational portal. Since past few years, BOLD has become a potential and central online platform for the researchers working in DNA barcoding fields. It has diverse data files which can be downloaded in different formats like Specimen data as XML or TSV, sequences as FASTA, Trace files as .ab1 or .scf and specimen details/sequences as XML or TSV formats. All essential features like data upload/download, public query, cross-referencing, user registration, help and analytical tools (Distribution Map Analysis, Taxon ID Tree, Distance Summary, sequence composition tools, etc.) make BOLD extremely perfect and user-friendly (Table 1). It is a *PostgreSql relational database* (www.postgresql.org) constructed using *Java, C ++, PHP* and can be accessed at http://www.boldsystems.org/. In order to further explore huge amount of global molecular data, the ‘Barcode of Life Data Portal’ (BDP; http://bol.uvm.edu) was constructed by *CBOL* using *PHP* (87). It is a central resource to access the information from BOLD as well as other public databases like NCBI GenBank. Thus, it bridges the gap between the DNA barcoding scientists and the biodiversity informatics researchers. It can also assist in accessing a vast array of approaches for exploration and cataloguing of the molecular data for DNA barcoding applications.

### Taxonomy/distribution related databases

Proper identification, classification, taxonomy, distribution and geographical information of species are the foremost things for biology, genetics, molecular studies, etc. Databases consisting of such information play a pivotal role in exploring, identifying and classifying species. The two such main databases are ‘Database of Insects and their Food Plants’ (DBIF) and ‘Database of Butterflies and Moth of the World’ (DBMW).

‘DBIF’ has been developed by *Biological Research Centre (BRC), England* as the main part of *National Biodiversity Network (NBN).* It is a database of invertebrates (including insects, silkworms) and their host plants with three search options- ‘Search invertebrates’, ‘Search host plants’ and ‘Search source’. Interactions for members of different families of Lepidoptera (∼7 butterfly families, ∼19 macro moth families and ∼42 micro moth families) are provided by this database. The output is displayed in a tabular format for the selected search and can be accessed through http://www.brc.ac.uk/dbif/homepage.aspx.

‘DBMW’ on the other hand, was developed by *Natural History Museum (NHM), UK* which catalogues around 32 000 generic names of world’s lepidopteran insects (butterflies and moths, including wild silkmoths). Information of around 88, 356 and 449 members of three families: Bombycidae, Saturniidae and Sphingidae can be retrieved from the database. The data can be searched or browsed through family, genus, species, classification or images and accessible at URL: http://www.nhm.ac.uk/our-science/data/butmoth/.

In addition to the above data resources, few other databases describing the morphological, ecological, taxonomical data of diverse live forms are available. These include: ‘Common Names of Insects Databases’ (CNIDB; http://www.entsoc.org/pubs/common_names), ‘Moths of Borneo’ (http://www.mothsofborneo.com/) under Host-Parasite database (http://www.nhm.ac.uk/research-curation/scientific-resources/taxonomy-systematics/host-parasites/), ‘Butterflies and Moths of North America’ (BAMONA, http://www.butterfliesandmoths.org/), ‘EPPO Global Database’ (https://gd.eppo.int), ‘Encyclopedia of Life’ (EOL) Database (http://www.eol.org), ‘Integrated taxonomic information system’ (ITIS) database (http://www.itis.gov/) and ‘INPN’ (http://inpn.mnhn.fr/).

### Pheromone databases

Since the discovery of sex pheromones in *B. mori* (1959), huge amount of data on pheromones and other signaling compounds of insects including some silkworms has been generated. These signaling chemicals are required for communication, interaction, behavior, sexual attraction, defense, behavioral activities, etc. ‘Pherobase’ is the world’s largest useful database of semiochemicals, i.e. pheromones and allelochemicals developed by *El-Sayed AM, HortResearch, Lincoln, New Zealand* in 2014 (88)*.* Presently, the database hosts around 30 000 entries, 3500 semiochemicals and 8000 organic compounds of not only insects but also plants (floral compounds), invasive species, etc. The classification was based on various criteria’s like functional groups, behavior, molecular weight, formula, etc. which can be browsed via taxa, family, genus and species. It has two inbuilt tools such as ‘Kovats calculator’ and ‘Formula generator’ to calculate kovats values and formula for specific ions respectively. It is a full-fledged database with facilities like regular updates, search, data submission, and sign in, etc. (Table 1). However, addition of download option will further enhance its applicability. This database is accessible through URL: http://www.pherobase.com/.

### Silk-based databases

Silk has evolved as a great source of economy during past decades as discussed earlier. Few databases like ‘Biomat_dBase’, ‘Spatio-temporal database of the Silk Road’ (SRDB) and ‘Silk Fabric Specification Database’ (SFSDB) have been reported by different groups to cover the available information related to silk (biomaterial, Silk Road and fabric characteristics).

‘Biomat_dBase’ has been constructed by *Indian Institute of Technology, Kharagpur, India* using *HTML/CSS, PHP* and *Javascripts*. This database combines the biomaterial information with main focus on natural biomaterials including silk (89). This information includes fabrication of silk into different matrices, applications in tissue engineering, regenerative medicine, etc. Although database URL: http://dbbiomat.iitkgp.ernet.in is mentioned in the publication but the link is not functional currently. Development and availability of this database will be helpful for the researchers working in the related fields in utilizing the existing resources and fabricating new biomaterials.

‘SRDB’ and ‘SFSDB’ are other similar databases mentioned in the publication but not accessible over internet. SRDB is a collaborative *SQL* server database developed by *Chinese Academy of Sciences, Surveying and Land Information Engineering of Central South University, China*, that contains the data related to Silk Road during the ancient times (90). The database mainly focuses on historical, field, geographical, remote sensing, thematic data of Han and Tang Dynasties. Accessibility of this database can act as a platform for combining both modern and archaeological technologies thus making a way towards development of a traditional archaeology. On the other hand, SFSDB was developed by *National Engineering Laboratory for Modern Silk* and *College of Textile and Clothing Engineering, Soochow University, China* (91, 92). The database constructed using *SQL Server 2000* and *Visual Basic.NET*, deals with the fabric specification information (fabric name, number, weaving information, etc.) and its analyses (calculation of cover tightness, fabric balance coefficient, fabric shrinkage, etc.). This database if made available will help the researchers in silk fabric designing and their development.

### Other web resources

During our search, we found few other databases (‘miRNEST’, ‘miRBase’ and ‘MEROPS’) which directly or indirectly contained information of few seri-resources.

‘miRNEST’ (current version: miRNEST v 2.0) is an integrated micro RNA database managed by *Laboratory of Functional Genomics, Adam Mickiewicz University, Poland.* Constructed using *HTML, CSS, PHP 5.2.11* and *MySQL 4.0.31*, it includes structure and targets of miRNA candidates of the silkworms and plants (93, 94). The miRNA sequences of silkworms namely *B. mori, S. cynthia* and host plants such as *P. americana, R. communis, Q. robur, C. papaya, M. esculenta, J. curcas* are included in this database. ‘miRBase’ is another miRNA resource managed by the *Griffiths-Jones lab* at the *Faculty of Life Sciences, University of Manchester, UK.* It is a *MySQL* database that comprises of information on miRNAs, their annotation and sequences of taxa like insects (e.g. *B. mori*), host plants (e.g. *R. communis, M. esculenta*) (95–98). ‘MEROPS’ (current Release 9.13), on the other hand, is a peptidase database designed and developed at *EMBL-European Bioinformatics Institute, Cambridge CB10 1SD, UK* using *MySQL.* Peptidases (proteolytic enzymes, proteases, proteinases) are the enzymes which degrade the proteins by hydrolyzing the peptide bonds and constitute around 2% of all the proteins in any organism. The database offers hierarchical classification and nomenclature of the peptidases, their substrates as well as inhibitors (99, 100).

The above three databases can be accessed freely through URL http://mirnest.amu.edu.pl, http://www.mirbase.org/ or as flat file from ftp://mirbase.org/pub/mirbase/ and http://merops.sanger.ac.uk, respectively.

The comparative analysis of all the databases discussed in the ‘Combined Databases’ section demonstrated that most of them are better designed than databases discussed in other sections and are equipped with most of the essential features. Many of these are highly cited: miRBase being the most cited database followed by BOLD, MEROPS and so on (Supplementary Table S2). The search visibility of all the databases is good and the databases are easy to access. However, few features like data update, analytical tools, help and user registration was not uniformly observed in all combined databases. Also, some accessibility issues related to non-functional URLs of a few databases (for eg- Biomat_dBase) were observed which require troubleshooting.

## Technology for data generation in sericulture field

Genomic technologies utilized in silkworm research involve structural to functional genomics. Structural genomics deals with three-dimensional structure of gene products. Functional genomics, on the other hand, is primarily concerned with the transcriptome, i.e. gene expression analysis and with proteome i.e. protein analysis (101). It deals with the expression profiling, usage of genome by the organism under physiological or developmental conditions, etc. This review covers a time frame of ∼12 years, from 2003 to 2015 (continued) (Figure 2). During this period, the technologies adapted for silkworm research have observed tremendous improvement (Table 2). The genome of *B. mori* was sequenced by BAC-end cloning and WGS sequencing (17). At present, sequencing technologies have progressed beyond old Sanger sequencing methodologies. NGS technologies are revolutionizing many areas of molecular biology such as genomics, transcriptomics, proteomics, etc. owing to their cost-effectiveness and unprecedented speed (102–106). Its main advantage is that gene discovery and expression profiling is possible through *de novo* assembly of short reads generated i.e. without any reference genome. Several NGS platforms such as Illumina/Solexa, ABI/SOLiD, 454/Roche, etc. provide broad opportunities for HT functional genomics e.g. insect chemical ecological studies such as pheromone production, reception, insect–plant interactions; genetic manipulation studies; proteomics studies, etc. (107). NGS has been applied for expression analyses of pheromone receptors, adverse effects of phoxim exposure in the *B. mori* (108). Further, functional complexity of seri-transcriptome necessitates the exploration of diverse fields which are not yet clarified. Studies reveal that the comparative transcriptomics through RNA-Seq technology unravel the genetic basis of silk production and strength, cocoon coloration, etc. among wild and domesticated silk moths (109, 110). Apart from the above ‘omics’ technologies, currently metabolomics, epigenomics and metagenomics are emerging as advanced approaches for studying the metabolome, epigenome and metagenome of the insects including silkmoths along with their resources (111). Changes in host metabolism, measurement of sugars, amino acids, redox agents or complex metabolite mixtures, epigenetic divergence and regulation through methylomics are few hidden areas which are yet to be applied in sericulture field (112, 113). Similarly, the metagenomic studies can reveal the microbial complexity in the gut of the insects (114, 115). Moreover, genetic studies based on transposon-mediated transgenesis and genome-editing technologies also have significant impact on genetic manipulations among silkworms (116).

**Table 2.** Technologies for data generation in sericulture field

Sl. No.	Sericultural research area	Technologies	References
1	Genomics	BAC-end sequencing filter, hybridization, fingerprinting, WGS, Transgenesis technology, comparative genomics, Linkage mapping, Sanger sequencing, Roche 454 Genome Sequencing, Pyrosequencing technology (454 GS-FLX), Combination of Illumina and 454, Illumina short-read sequencing, de novo seq, exome seq, targeted seq, Microarray based and genome wide association studies (GWAS)	(8, 104, 107)
2	Proteomics	SDS-PAGE, Tandem MS (Tandem Mass Spectroscopy), MALDI MS, two-dimensional gel electrophoresis (2-DE), protein microarray	(108,117, 118)
3	Transcriptomics	High-throughput RNA sequencing technology (RNA-Seq), Comparative transcriptome analysesTotal RNA and mRNA sequencing, Targeted RNA Sequencing, Small RNA and Non-coding RNA Sequencing, Serial analysis of gene expression (SAGE)	(107, 109, 110)
4	Metabolomics	MS –based system with GC (Gas chromatography) and LC (Liquid Chromatography) for initial separation, NMR analysis of crude extracts and its direct examination by MS	(113, 119)
5	Epigenomics	Methylation sequenicng, Illumina high-throughput bisulfite sequencing (MethylC-Seq), ChiP Sequencing, Ribosome Profiling, Pyrosequencing technology	(107, 112)
6	Metagenomics	Metagenomics: Amplicon seq (16S rRNA), Shotgun Sequencing	(113, 115)
7	Metatrancriptomics	Metatrancriptomics: Functional study of microbial populations Illumina RNA-Seq	(113, 115)
8	Genetic Manipulation	Genetic technologies- Transposon based or genome-editing technologies	(116)

## Outcome of the study: SeriPort

The review of literature as well as exploration of cyberspace for all available data resources culminated into a huge amount of seri-database related information. As mentioned in the database descriptions or demerits above, some of these databases are not easily available to users due to their minimal turn-up during common searches using popular search engines. Again, some of the related web-resources are not published in literature and hence, are not known to the public. In order to address these issues, we have created an HTML based web portal which can act as a common platform for all kinds of data available on sericulture in the internet. The portal has been named ‘SeriPort’ and is available at http://seriport.in/ (Supplementary Figure S1). The workflow for the construction of SeriPort is schematically represented in Supplementary Figure S2. The database for SeriPort was designed using Basic HTML 5 and CSS 3 for front-end and PhpMyAdmin and MySQL 5 for the back end (Supplementary Figure S3). The working language of website is PHP and HTML 5. The connectivity between front end and back end was done by using PHP. The central data in SeriPort, i.e. databases on silkworms, host plants, pest and pathogens, etc. were categorized in the portal in a similar manner to that of the present review. However, a separate webpage was created for each database which consisted of a short description and its web link. Apart from that, the portal also has a webpage on relevant references on these databases. The main features of SeriPort include a user-friendly and dynamic user interface, in-built search engine and options for data download and submission. SeriPort, which is an outcome of this review, is expected to serve as a supportive portal between general users and the niche occupied by sericulture in the internet.

## Conclusion

In this review, we have highlighted the databases which currently provide information on the biotic components of a silkworm’s ecological niche. Silkworm thrives on plant leaves and co-inhabits its host plant with numerous other insects and micro-organisms which may act as its pest or pathogen. The efforts to understand silkworm or its interactions with other organisms have generated a plethora of information which has been converted into different types of electronic databases. The applicability as well as advantages and limitations of these databases have been previously discussed. It has been observed that problems related to data update, public data submission and incorporation, data analysis tools were common among the databases. Most of these drawbacks can be dealt with proper database architecture and programming. If we attempt to measure the usefulness of these databases, the citation count per publication can be taken into account. The database related articles have been cited ∼13 553 times (on 11 March 2016) which is a significant value. Amongst the databases, the highly cited ones are SilkBase, BmMDB, SilkDB, SilkDB v2.0, Phytozome, PlantTFDB, Jatropha genome database, miRBase, BOLD and MEROPS (Supplementary Table S2).

The importance of seri-databases cannot be emphasized more. They can play crucial roles in conservation of the silkworm species, especially the wild varieties. For example, *A. assamensis* is a semi-domesticated silkworm which is endemic to North-Eastern part of India, mainly due to the climatic and environmental conditions of the place. WildSilkbase which provides the complete EST set of *A. assamensis* can assist us in understanding the functional part of its genome. This might help in engineering the organism to be able to survive in unfavorable conditions. Similarly, plant databases providing information on gene and protein sequences, diseases, etc. will facilitate the conservation of host plants, which is further important for the conservation of silkworms.

Among other scientific benefits are studies on complex interactions among silk moths, host plants and microbes as a model system to understand ecological balance; studies to understand genetics of silk materials from different silkworms; development of SNP-based molecular markers to aid in species differentiation; host plant improvement via genetic engineering; development of effective and cheap methodologies for detection and elimination of pest and pathogen infestations in silkworms and host plants; etc. Also, proper documentation of huge information in a secure but accessible place is another reason to have more seri-related databases in the future. Implementation of informatics and HT technologies can aid in this regards to a great extent. From an economic standpoint, we believe that the databases can indirectly influence the economy of a region which is dependent on silkworms or their host plants for daily income. India and China are some of the sericulture-intensive countries in the world providing employment to around 9 million people, according to International Sericultural Commission. We have already witnessed the devastating consequences of Colony Collapse Disorder of honey bees in Europe. If an epidemic of pest infestation is to occur in these places, a huge number of families will be hit by it. These seri-bioresource databases will facilitate more studies on understanding the likeliness of such epidemics and development of future strategies to tackle them. Moreover, seri-bioresources will be highly benefited by the databases and it is imperative that the process of developing more databases goes on. New databases can be created for pest and pathogens, geographical locations, silkworm and host plant disease statistics, taxonomy, compounds, pathways, and so on.

SeriPort, the web-portal which is an outcome of this review can help general users in finding the sericulture-related databases over the internet in a more effective manner. Also, this portal will shed light on useful databases which are not known or seldom accessed due to invisibility in top search results.

## Supplementary data

Supplementary data are available at Database Online.

## Acknowledgements

DS, HC and DK express gratitude to MHRD (Government of India) for financial support in the form of fellowship. They also thank Institutional Biotech Hub (Project BT/04/NE/2009) established under Department of Biotechnology (DBT), Government of India for providing computational facility to carry out the research work.

## Funding

Department of Biotechnology, Government of India, New Delhi for supporting the research through U-Excel Project (Sanction Order No. BT/411/NE/U-Excel/2013 dated 06.02.2014).

*Conflict of interest*. None declared.

## Websites URL

EPPO. (2016). EPPO Global Database (available online). https://gd.eppo.int

Integrated Taxonomic Information System (ITIS) http://www.itis.gov

Common Names of Insects and Related Organisms http://www.entsoc.org/pubs/common_names

Butterflies and moths of North America http://www.butterfliesandmoths.org/

INPN http://inpn.mnhn.fr/

Butterflies and Moths of the World http://www.nhm.ac.uk/our-science/data/butmoth/

Database of Insects and their Food Plants http://www.brc.ac.uk/dbif/homepage.aspx

Chinese Cassava Genome Database http://www.cassava-genome.cn/

CassavaBase http://www.cassavabase.org/

Cassava Genome Database http://cassava.igs.umaryland.edu/cgi-bin/index.cgi

Databases Developed and Maintained at Bioinformatics Centre, CSRTI, Mysore (http://www.btismysore.in/dbase.html)

Castor Database http://www.tnaugenomics.com/castor/index.php

SilkwormBase http://www.shigen.nig.ac.jp/silkwormbase/about_kaiko.jsp

ABURAKO database-The world of silkworm larval translucent skin mutants (http://cse.nias.affrc.go.jp/natuo/en/aburako_top_en.htm)

Database of Bombyx mutant photographs, IGB Lab, Univ. Tokyo (http://papilio.ab.a.u-tokyo.ac.jp/genome/)

Moths of Borneo (http://www.mothsofborneo.com/)

## Supplementary Material

Supplementary Data
