# Supplementary material for: A comprehensive view of the web-resources related to sericulture
Source: Database (Oxford). 2016 Jun 15;2016:baw086. doi: 10.1093/database/baw086 (PMC4909305; doi:10.1093/database/baw086)
Supplement: Supplementary Data [file supp_2016_baw086_index.html]

Supplementary Data 

# A comprehensive view of the web-resources related to sericulture

## Supplementary Data

files

- Supplementary Data - zip file
