# Supplementary material for: A comprehensive view of the web-resources related to sericulture
Source: Database (Oxford). 2016 Jun 15;2016:baw086. doi: 10.1093/database/baw086 (PMC4909305; doi:10.1093/database/baw086)
Supplement: Supplementary Data [file supp_baw086_suppl_data.zip › Table S2-Citation_Revised.docx]

**Table S2.** Citation analysis of Seri related databases and their consecutive research articles (Data obtained from Scopus, Google Scholar and Web of Science on **11-03-2016**)

**A. SILKWORM DATABASES**

| **Sl. No.** | **Database Name** | **Year of publication and Journal Name** | **Article Title** | **Scopus** | **Google Scholar** | **Web of Science** | **Total** | **Average** |
| --- | --- | --- | --- | --- | --- | --- | --- | --- |
| 1 | SilkBase | 2003  (*Proceedings of the National Academy of Sciences*) | The construction of an EST database for *Bombyx mori* and its application | 200 | 252 | 222 | 674 | **225** |
| 2 | BmMDB | 2007  (*BMC* *Genome Biology*) | Microarray-based gene expression profiles in multiple tissues of the domesticated silkworm,  *Bombyx mori* | 168 | 220 | 190 | 578 | **193** |
| 3 | SilkDB | 2009  (*Nucleic Acids Research*) | SilkDB v2. 0: a platform for silkworm (*Bombyx mori*) genome biology | 121 | 158 | 102 | 381 | **127** |
| 4 | SilkDB | 2005  (*Nucleic Acids Research*) | SilkDB: a knowledgebase for silkworm biology and genomics | 111 | 140 | 95 | 346 | **115** |
| 5 | Butterfly  Base | 2008  (*Nucleic Acids Research*) | ButterflyBase: a platform for lepidopteran genomics | 61 | 78 | 59 | 198 | **66** |
| 6 | Kaikobase | 2009  (*BMC Genomics*) | KAIKObase: an integrated silkworm genome database and data mining tool | 56 | 68 | 57 | 181 | **60** |
| 7 | InSatDB | 2007  (*Nucleic Acids Research*) | InSatDb: a microsatellite database of fully sequenced insect genomes | 24 | 36 | 30 | 90 | **30** |
| 8 | WildSilkbase | 2008  (*BMC Genomics*) | WildSilkbase: an EST database of wild silkmoths | 27 | 31 | 27 | 85 | **28** |
| 9 | SilkSatDB | 2005  (*Nucleic Acids Research*) | SilkSatDb: a microsatellite database of the silkworm, *Bombyx mori* | 19 | 25 | 20 | 64 | **21** |
| 10 | SilkTransDB | 2012  (*PloS One*) | Transcriptome analysis of the silkworm  (*Bombyx mori*) by high-throughput RNA sequencing | 16 | 20 | 17 | 53 | **18** |
| 11 | BmTEdb | 2013  (*Database*) | BmTEdb: a collective database of transposable elements in the silkworm genome | 6 | 10 | 1 | 17 | **6** |
| 12 | Kaiko2DDB | 2006  *(Journal of Electrophoresis)* | Draft of silkworm proteome database | NF | 7 | NF | 7 | **2** |
| 13 | Silkworm Gene Resources Database | 2010 (*Bioinformation*) | An integrated database for the enhanced identification of silkworm gene resources | NF | 2 | 2 | 4 | **1** |
| 14 | SilkPPI | 2014 (*Bioinformation*) | In silico identification of protein-protein interactions in Silkworm, *Bombyx mori* | NF | 2 | 2 | 4 | **1** |
| 15 | iPathDB | *2014*  *(Database*) | iPathCons and iPathDB: an improved insect pathway construction tool and the database. *Database* | 0 | 1 | 0 | 1 | **0** |

**B. HOST PLANT DATABASES**

| **Sl. No.** | **Database Name** | **Year of publication and Journal Name** | **Article Title** | **Scopus** | **Google Scholar** | **Web of Science** | **Total** | **Average** |
| --- | --- | --- | --- | --- | --- | --- | --- | --- |
| 1 | Phytozome | 2012  (*Nucleic Acids Research*) | Phytozome: a comparative platform for green plant genomics | 590 | 792 | 528 | 1910 | **637** |
| 2 | Plant Transcription Factor Database (PlantTFDB) | 2011  (*Nucleic Acids Research*) | PlantTFDB 2.0: update and improvement of the comprehensive plant transcription factor database | 153 | 197 | 138 | 488 | **163** |
| 3 | Jatropha genome database | 2011  (*DNA Research*) | Sequence analysis of the genome of an oil-bearing tree, *Jatropha curcas* L. | 128 | 180 | 110 | 418 | **139** |
| 4 | PlantGDB | 2008  (*Nucleic Acids Research*) | PlantGDB: a resource for comparative plant genomics | 110 | 148 | 98 | 356 | **119** |
| 5 | Plant Transcription Factor Database (PlantTFDB) | 2014  (*Nucleic Acids Research*) | PlantTFDB 3.0: a portal for the functional and evolutionary study of plant transcription factors | 97 | 140 | 95 | 332 | **111** |
| 6 | Plant Transcription Factor Database (PlantTFDB) | 2008  (*Nucleic Acids Research*) | PlantTFDB: a comprehensive plant transcription factor database | 94 | 140 | 86 | 320 | **107** |
| 7 | PLAZA | 2009  (*The Plant Cell*) | PLAZA: a comparative genomics resource to study gene and genome evolution in plants | 143 | 3 | 129 | 275 | **92** |
| 8 | PlantGDB | 2005  (*Plant Physiology*) | Comparative plant genomics resources at PlantGDB | 72 | 97 | 66 | 235 | **78** |
| 9 | HOSTS | 2010 *(The Natural History Museum in London)* | HOSTS―a Database of the World's Lepidopteran Hostplants | NF | 175 | NF | 175 | **58** |
| 10 | ChromDB- The Chromatin Database | 2008  (*Nucleic Acids Research*) | ChromDB: the chromatin database | 44 | 71 | 38 | 153 | **51** |
| 11 | PLAZA | 2015  (*Nucleic Acids Research*) | PLAZA 3.0: an access point for plant comparative genomics | 20 | 28 | 18 | 66 | **22** |
| 12 | Quercus Portal | 2015 *(BMC Genomics)* | The oak gene expression atlas: Insights into Fagaceae genome evolution and the discovery of genes regulated during bud dormancy release | 4 | 12 | 4 | 20 | **6** |
| 13 | CastorDB: a comprehensive knowledgebase for *Ricinus communis* | 2011  *(BMC Research Notes)* | CastorDB: a comprehensive knowledge base for *Ricinus communis* | 5 | 6 | 5 | 16 | **5** |
| 14 | Quercus Portal | 2016  *(Molecular Ecology Resources)* | Decoding the oak genome: public release of sequence data, assembly, annotation and publication strategies | 0 | 7 | 1 | 8 | **2** |
| 15 | MorusDB: a resource for mulberry genomics and genome biology | 2014 (*Database*) | MorusDB: a resource for mulberry genomics and genome biology | 0 | 4 | 0 | 4 | **1** |
| 16 | MulSatDB | 2014  (*Trees*) | MulSatDB: a first online database for mulberry microsatellites | 0 | 1 | 1 | 2 | **0** |
| 17 | CPR-DB | 2014 *(Genetics and Genomics of Papaya)* | Papaya Repeat Database | NF | 1 | NF | 1 | **0** |

**C. PEST/PATHOGEN DATABASES**

| **Sl. No.** | **Database Name** | **Year of publication and Journal Name** | **Article Title** | **Scopus** | **Google Scholar** | **Web of Science** | **Total** | **Average** |
| --- | --- | --- | --- | --- | --- | --- | --- | --- |
| 1. | SilkPathDB | 2013 (*BMC Genomics*) | Comparative genomics of parasitic silkworm microsporidia reveal an association between genome expansion and host adaptation | 35 | 39 | 48 | 122 | **41** |

**D. COMBINED DATABASES**

| **Sl. No.** | **Database Name** | **Year of publication and Journal Name** | **Article Title** | **Scopus** | **Google Scholar** | **Web of Science** | **Total** | **Average** |
| --- | --- | --- | --- | --- | --- | --- | --- | --- |
| 1 | miRBase | 2008  (*Nucleic Acids Research*) | miRBase: Tools for microRNA genomics | 2400 | 3275 | 2200 | 7875 | **2625** |
| 2 | miRBase | 2006  (*Nucleic Acids Research*) | miRBase: microRNA sequences, targets and gene nomenclature | 2089 | 3103 | 2056 | 7248 | **2416** |
| 3 | miRBase | 2011  (*Nucleic Acids Research*) | miRBase: integrating microRNA annotation and deep-sequencing data | 1704 | 2351 | 1538 | 5593 | **1864** |
| 4 | BOLD | 2007  *(Molecular Ecology Notes)* | BOLD: The Barcode of Life Data System (http://www. barcodinglife. org) | 1292 | 1560 | 1365 | 4217 | **1406** |
| 5 | miRBase | 2014  (*Nucleic Acids Research*) | MiRBase: Annotating high confidence microRNAs using deep sequencing data | 524 | 843 | 520 | 1887 | **629** |
| 6 | MEROPS | 2010  (*Nucleic Acids Research*) | MEROPS: the peptidase database | 478 | 622 | 403 | 1503 | **501** |
| 7 | MEROPS | 2012  (*Nucleic Acids Research*) | MEROPS: the database of proteolytic enzymes, their substrates and inhibitors | 429 | 572 | 378 | 1379 | **460** |
| 8 | MEROPS | 2008  (*Nucleic Acids Research*) | MEROPS: the peptidase database | 408 | 569 | 385 | 1362 | **454** |
| 9 | MEROPS | 2006  (*Nucleic Acids Research*) | MEROPS: the peptidase database | 383 | 579 | 368 | 1330 | **443** |
| 10 | MEROPS | 2014  (*Nucleic Acids Research*) | MEROPS: the database of proteolytic enzymes, their substrates and inhibitors | 178 | 253 | 152 | 583 | **194** |
| 11 | miRNEST | 2012  (*Nucleic Acids Research*) | miRNEST database: an integrative approach in microRNA search and annotation | 21 | 27 | 19 | 67 | **22** |
| 12 | EOL DB | 2014  (*Biodiversity Data Journal*) | The encyclopedia of life v2: providing global access to knowledge about life on Earth | NF | 17 | 0 | 17 | **6** |
| 13 | miRNEST | 2014  (*Nucleic Acids Research*) | miRNEST 2.0: a database of plant and animal microRNAs | 3 | 7 | 4 | 14 | **5** |
| 14 | EOL DB | 2009  (*BioScience*) | The encyclopedia of life: describing species, unifying biology | 3 | 3 | 2 | 8 | **3** |
| 15 | Biomat _dBase | *2012*  *(Open Tissue Engineering and Regenerative Medicine Journal)* | Biomat _dBase: A Database on Biomaterials | 0 | 1 | NF | 1 | **0** |
| 16 | Spatio-temporal DB of Silk Road | *2014*  *(IOP Conference Series: Earth and Environmental Science)* | The construction of the spatio-temporal database of the ancient Silk Road within Xinjiang province during the Han and Tang dynasties | 0 | 0 | NF | 0 | **0** |
| 17 | Silk Fabric Specification DB | *2012*  *(Advanced Materials Research)* | Study on Selvage Warp Coefficient Based on Silk Fabric Specifications Database | 0 | 0 | NF | 0 | **0** |

**NOTE:** NF stands for NOT FOUND
