# Supplementary material for: A comprehensive view of the web-resources related to sericulture
Source: Database (Oxford). 2016 Jun 15;2016:baw086. doi: 10.1093/database/baw086 (PMC4909305; doi:10.1093/database/baw086)
Supplement: Supplementary Data [file supp_baw086_suppl_data.zip › Annexure 1.docx]

**Annexure 1**

**Contents**

1. **Introduction**
2. **Silkworm Databases**
   1. **Nucleotide Databases**
      1. **Silkworm Genome Databases**
      2. **Silkworm Gene Expression Databases**
      3. **Microsatellite Databases**
      4. **Silkworm Mutant Databases**
      5. **Transposable elements (TEs) Databases**
      6. **Other web-resources**
   2. **Protein Databases**
   3. **Silkworm Genetic Resource Databases**
   4. **Insect Pathway Databases**
3. **Silkworm Host Plant Databases**
   1. **Databases of Mulberry**
   2. **Databases of Castor**
   3. **Databases of Papaya**
   4. **Databases of *Jatropha***
   5. **Databases of Cassava**
   6. **Databases of *Quercus***
   7. **Other Generalized Plant Databases**
4. **Pest and Pathogen Databases**
5. **Combined Databases**
   1. **Barcode Databases**
   2. **Taxonomy/Distribution related Databases**
   3. **Pheromone Databases**
   4. **Silk-based Databases**
   5. **Other web resources**
6. **Technology for Data Generation in Sericulture Field**
7. **Outcome of the Study: SeriPort**
8. **Conclusion**
9. **Acknowledgement**
10. **References**
