# Supplementary material for: A comprehensive view of the web-resources related to sericulture
Source: Database (Oxford). 2016 Jun 15;2016:baw086. doi: 10.1093/database/baw086 (PMC4909305; doi:10.1093/database/baw086)
Supplement: Supplementary Data [file supp_baw086_suppl_data.zip › Annexure 2.docx]

**Annexure 2**

**List of Abbreviations***

**API:** Application programming interface

**AS:** Alternative splicing

**BAMONA:** Butterflies and Moths of North America

**BDP:** Barcode of Life Data Portal

**BGI:** Beijing Genomics Institute

**BmMDB:** *Bombyx mori* Microarray Database

**BmTEdb:** Transposable elements database for *B. mori*

**BOLD:** Barcode of Life Data System

**BRC:** Biological Research Centre

**CastorDB:** A comprehensive knowledgebase DB for *R. communis*

**CBOL:** Consortium for the Barcode of Life

***CCDB:*** *Chinese Cassava Genome Database*

**CDFD:** Centre for DNA Fingerprinting and Diagnostics

***CGDB:*** *Cassava Genome Database*

**ChromDB:** The Chromatin Database

***CNIDB:*** *Common Names of Insects Databases*

**CPR-DB:** Papaya Repeat Database

**CSR&TI:** Central Sericulture Research and Training Institute

**DB:** Database

**DBIF:** Database of Insects and their Food Plants

***DBMP:*** *Database of Bombyx mutant photographs*

***DBMW:*** *Database of Butterflies and Moth of the World*

**EOL:** Encyclopedia of Life

**EST DB:** Expressed Sequence Tag Database

**ESTs:** Expressed sequence tags

**FISH:** Fluorescence in situ hybridization

**HCN:** High copy number

**HOSTS:** a Database of the World's Lepidopteran Host plants

**HT:** High-throughput

**INRA:** Institut National de la Recherche Agronomique

**iPathDB:** Insect Pathway Database

**ISGSC:** International silkworm genome sequencing consortium

**ISSB:** Institute of Sericulture and Systems Biology

**ISSR:** Inter simple sequence repeat

**ITIS:** Integrated taxonomic information system

**MEROPS:** the peptidase database

**miRBase:** The microRNA database

**miRNEST:** An integrative microRNA resource

**MorusDB:** Morus Genome Database

**MulDis:** A Comprehensive Mulberry Disease and Pest Database

**MulSatDB:** Mulberry Microsatellite Database

**NAAS:** National Academy of Agricultural Science

**NBN:** National Biodiversity Network

**NBRP:** National BioResource Project

**NGS:** Next generation sequencing

**NHM:** Natural History Museum

**NIAS:** National Institute of Agrobiological Sciences

**OGSs:** official gene sets

**Papaya-DB:** Papaya Genomic Resources Online

**PDB:** Protein Data Bank

**PGDD:** Plant Genome Duplication Database

**PlantGDB:** Resources for Comparative Plant Genomics

**PlantTFDB:** Plant Transcription Factor Database

**RDBMS:** Relational Database Management System

**SAGE:** Serial analysis of gene expression

***SFSDB:*** *Silk Fabric Specification Database*

***SGRDB:*** *Silkworm Gene Resources database*

**SGRP:** Silkworm Genome Research Program

**SilkDB:** Silkworm Knowledgebase

**SilkPathDB:** Silkworm Pathogen Database

**SilkPPI:** Silkworm Protein- Protein Interaction database

**SilkProt:** Annotated protein database of silkworm

**SilkSatDb:** Silkworm Microsatellite Database

**SilkTF:** Silkworm Transcription Factor Database

**SilkTransDB:** Silkworm Transcriptome Database

**SKLSGB:** State Key Laboratory of Silkworm Genome Biology

***SRDB:*** *Spatio-temporal database of the Silk Road*

**SSR:** Simple Sequence Repeat

**SWU:** Southwest University

**TE:** Transposable element

**TNAU:** Tamil Nadu Agricultural University

**TRs:** Tandem repeats

**WGS:** Whole genome shotgun

**YRCH:** Yethapur *Ricinus communis* Hybrid

*(Abbreviations in italics were defined by the authors)*
