# Supplementary figures and images for: A comprehensive view of the web-resources related to sericulture
Source: Database (Oxford). 2016 Jun 15;2016:baw086. doi: 10.1093/database/baw086 (PMC4909305; doi:10.1093/database/baw086)

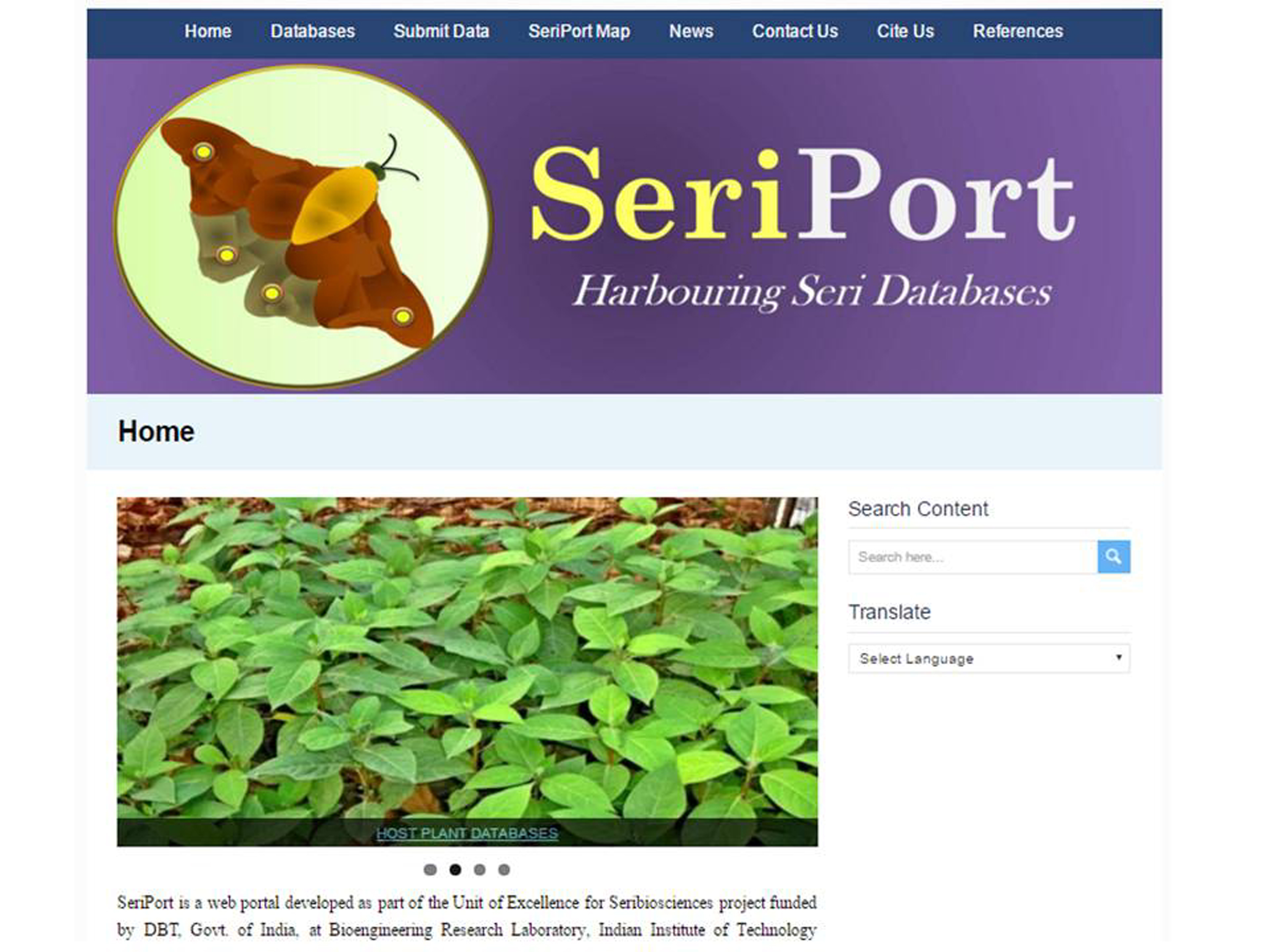

Supplement: Supplementary Data [file supp_baw086_suppl_data.zip › Figure S1-Home Page of SeriPort_Revised_baw086.jpg]

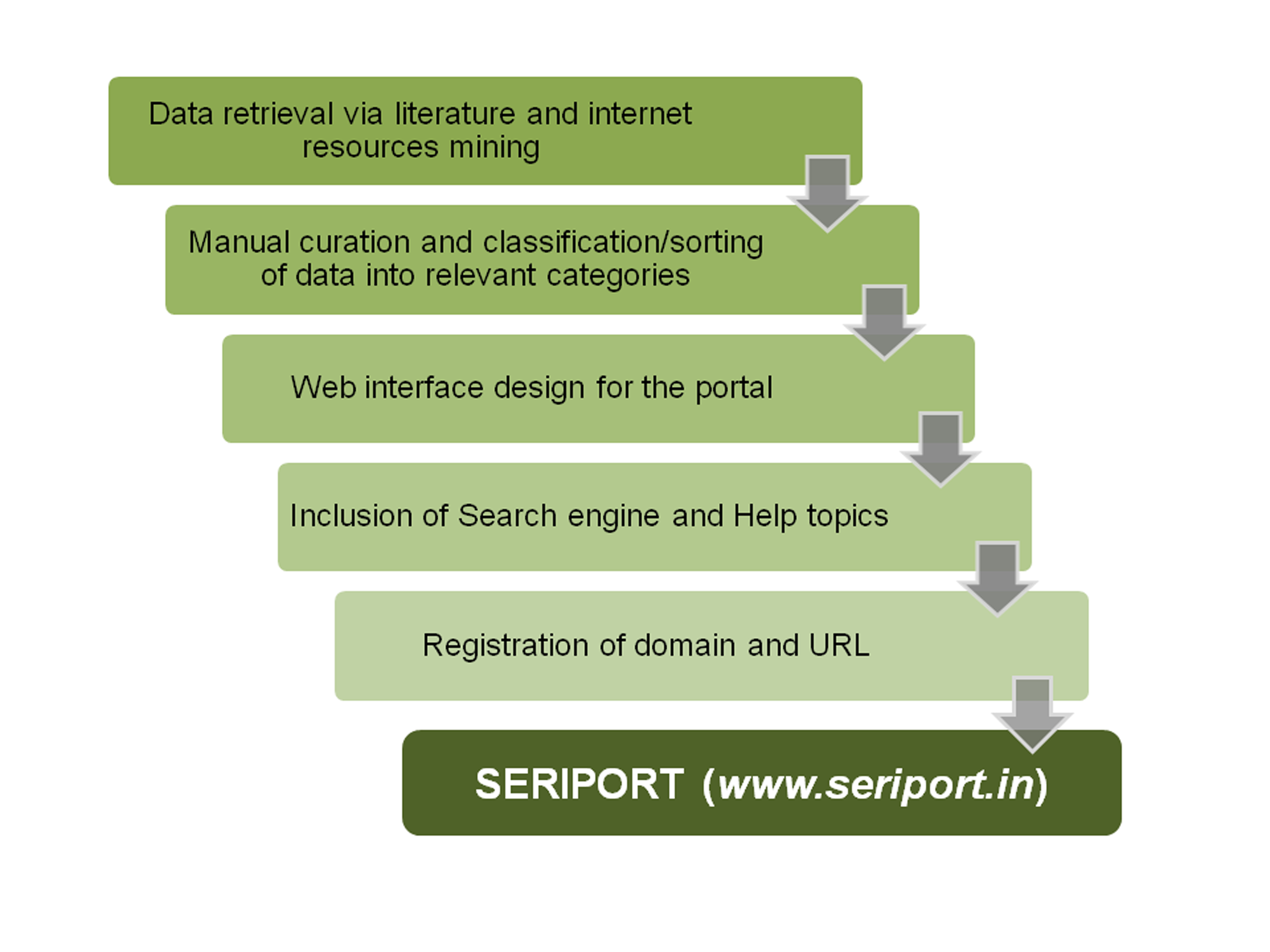

Supplement: Supplementary Data [file supp_baw086_suppl_data.zip › Figure S2-Workflow for the construction of SeriPort_Revised.tif]

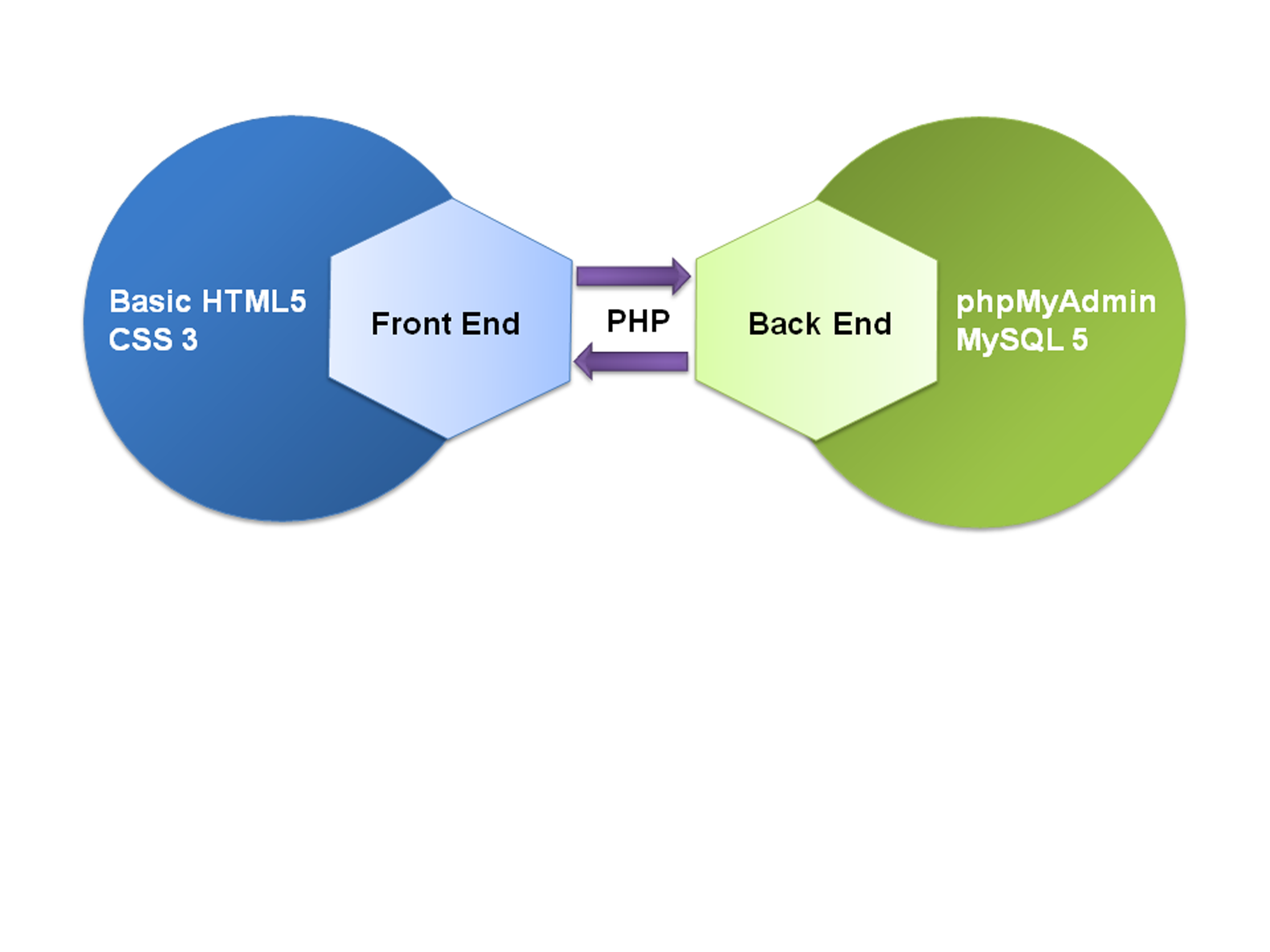

Supplement: Supplementary Data [file supp_baw086_suppl_data.zip › Figure S3-Schematic of database design for SeriPort_Revised.tif]

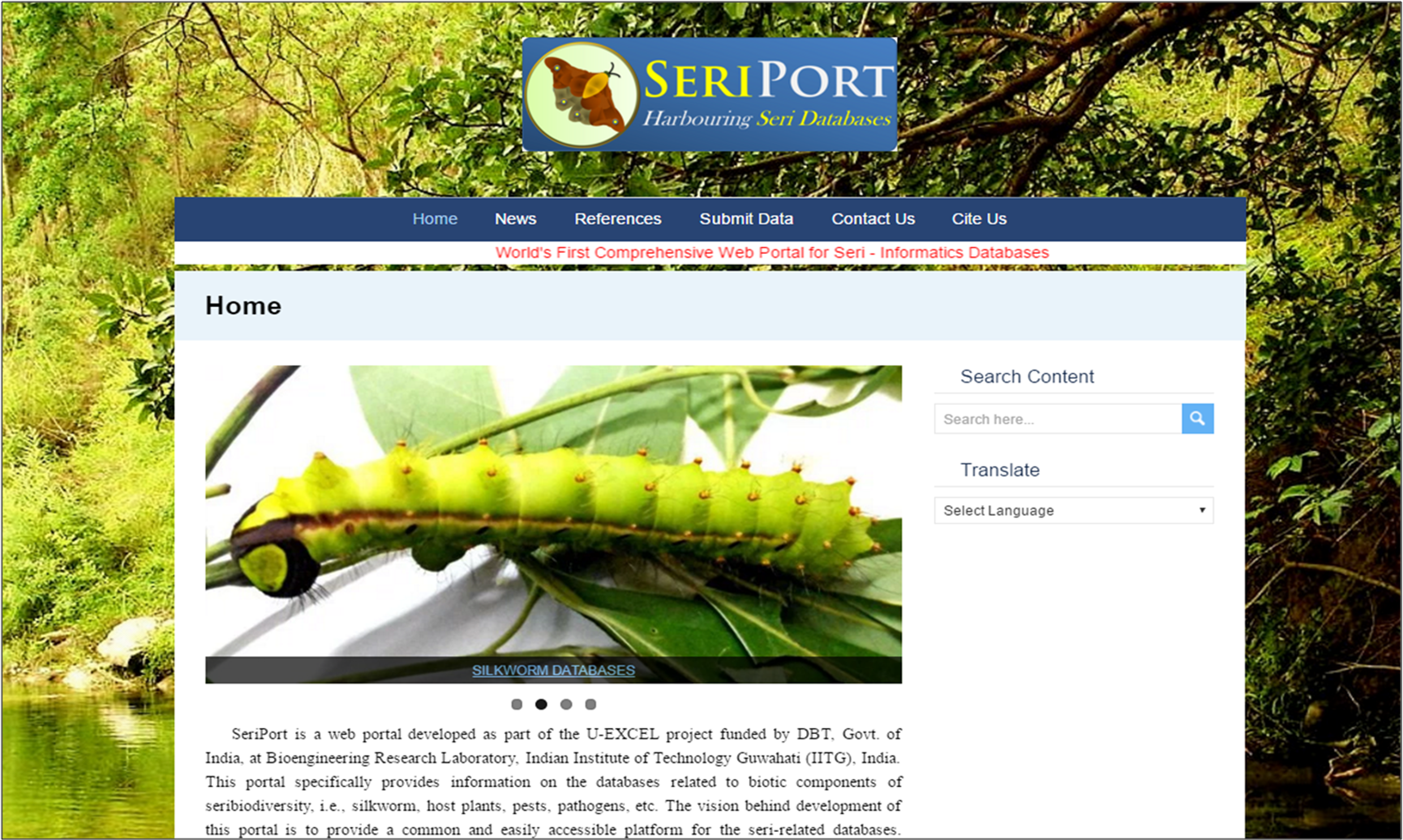

Supplement: Supplementary Data [file supp_baw086_suppl_data.zip › Figure S1-Home Page of SeriPort_Revised.tif]
